# Polycondensation Resins by Flavonoid Tannins Reaction with Amines

**DOI:** 10.3390/polym9020037

**Published:** 2017-01-25

**Authors:** Francisco-Jose Santiago-Medina, Antonio Pizzi, Maria Cecilia Basso, Luc Delmotte, Alain Celzard

**Affiliations:** 1 LERMAB, University of Lorraine, 27 rue Philippe Seguin, 88000 Epinal, France; francisco-jose-santiago-medina@univ-lorraine.fr (F.-J.S.-M.); cecilia-c-c@hotmail.com (M.C.B.); 2 Department of Physics, King Abdulaziz University, 21589 Jeddah, Saudi Arabia; 3 Institut de Science des Matériaux de Mulhouse, CNRS LRC 7228, 15, rue Jean Starcky, BP 2488, 68057 Mulhouse Cedex, France; luc.delmotte@uha.fr; 4 Institute Jean Lamour, University of Lorraine, 27 rue Philippe Seguin, 88000 Epinal, France; alain.celzard@univ-lorraine.fr

**Keywords:** flavonoid tannin amines reactions, oligomers distribution, resins, MALDI-ToF, CP-MAS ^13^C NMR

## Abstract

Reaction of a condensed flavonoid tannin, namely mimosa tannin extract with a hexamethylene diamine, has been investigated. For that purpose, catechin was also used as a flavonoid model compound and treated in similar conditions. Solid-state cross-polarisation/magic-angle spinning (CP-MAS) carbon 13 nuclear magnetic resonance (^13^C NMR) and matrix assisted laser desorption ionisation time of flight (MALDI-ToF) mass spectroscopy studies revealed that polycondensation compounds leading to resins were obtained by the reaction of the amines with the phenolic hydroxy groups of the tannin. Simultaneously, a second reaction leading to the formation of ionic bonds between the two groups occurred. These new reactions have been shown to clearly lead to the reaction of several phenolic hydroxyl groups, and flavonoid unit oligomerisation, to form hardened resins.

## 1. Introduction

Condensed polyflavonoid tannin extracts are mostly composed of flavan-3-ols repeating units and smaller fractions of polysaccharides and simple sugars [1]. The repeating units are linked to each other by C4–C6 or C4–C8, the former predominating in tannins in which fisetinidin (resorcinol A-ring; catechol B-ring) and robinetinidin (resorcinol A-ring; pyrogallol B-ring) are the predominant repeating units. While the reactions of these natural oligomeric materials have been used extensively to give polycondensates with aldehydes [2], even reactions of self-condensation have been studied and shown to lead to useful physically and chemically crosslinked networks [3,4,5,6,7].

Reactions of amination of phenols are well known, with the original approach to this reaction being by metal catalysis [8,9]. More recently, direct amination of phenols without the use of a metal catalyst has come to the fore and gained interest and importance [10,11].

Reactions of amination of flavonoid tannins to convert part of the phenolic hydroxyl groups of the B-ring to –NH_2_ have been studied before [12,13,14]; they have been exclusively with ammonia, although the literature on this is limited to just three articles [12,13,14]. There appear to be no references on the reaction of amines, diamines, or polyamines with condensed tannins in the relevant literature. The oldest of the articles on the ammonia reaction with flavonoid tannins was aimed to even more efficiently bind formaldehyde gas emitted from tannin adhesive resins for wood panels [12]. In this study, amination of pyrogallol B-rings of condensed tannins to form 4′-amino-3′,5′-dyhydroxybenzene type B-rings by NH_3_ treatment was described. The amination of the pyrogallol B-ring by NH_3_/water is a regioselective amino-substitution of phenolic hydroxyl groups and proceeds under relatively mild conditions without a catalyst [12,13,14]. While early reports indicated that only one hydroxy group of the flavonoid B-ring is aminated [12], later work showed that multiamination also proceeds with relative ease [14], mainly to prepare carbonized materials richer in nitrogen. These amination reactions, however, did not appear to lead to long oligomers and finally to crosslinked resins. To obtain then polycondensation resins without the use of any aldehyde, the reaction of condensed tannins with a diamine were investigated in the work presented here. The aim of this work was to obtain thermoset resins having a very rapid initial gelling either (i) by using only amines and polyamines as the only hardener; or (ii) to use two hardeners, of which the amine was the one giving just the initial immobilization of the resin—for example, for spray-projected coatings to avoid initial running down on vertical walls.

The reactions of diamines with condensed tannin to form resins were investigated here, using first catechin as a flavonoid model compound, followed by the same reactions on a condensed tannin analysed by extensive MALDI-ToF spectroscopy and solid-state cross-polarisation/magic-angle spinning (CP-MAS) ^13^C NMR studies. The findings are presented in this article.

## 2. Materials and Methods

### 2.1. Materials and Reactions

**Figure 1:**
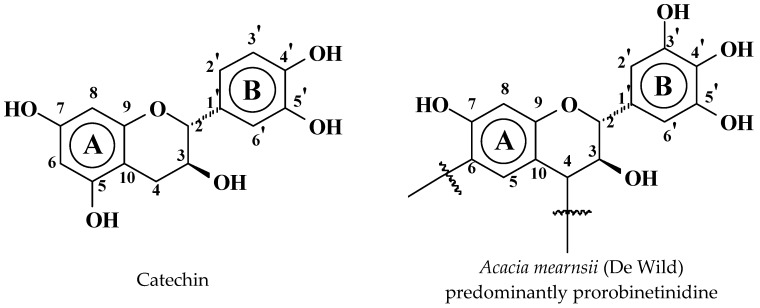
Structure of catechin and structure of the predominant flavonoid unit of mimosa tannin, robinetinidin, with its predominant C4 and C6 sites linked C4–C6 to other flavonoid units.

Catechin crystals (purity > 98%, high pressure liquid chromatography (HPLC) quality) was supplied by Sigma Aldrich (St. Louis, MO, USA) as (+)-catechin hydrate. Tannin extract was a commercial product, namely mimosa tannin extracted from barks of *Acacia mearnsii* (De Wild), supplied by SilvaChimica (St Michele Mondovi, Italy). It contained 80%–82% of actual phenolic flavonoid materials, 4%–6% of water, 1% of amino and imino acids, with the remainder being monomeric and oligomeric carbohydrates, generally broken pieces of hemicelluloses (see Figure 1).

From these two compounds, the following experiments have been carried out.

The samples were prepared as follow:

(1) Catechin (0.5 g) was mixed with 0.5 g of hexamethylenediamine (HMDA) (70% solution in water). Three samples were prepared with the proportions above. Then, each sample was reacted in an oven at 65, 100, and 185 °C overnight, respectively.
(2) Catechin (0.5 g) was mixed with 0.5 g of HMDA (70% solution in water) and 0.15 g of a 65 wt % aqueous solution of *p*-toluenesulfonic acid (pTSA). Again, three samples were prepared with the proportions above, and they were reacted in an oven at 65, 100, and 185 °C overnight, respectively.
(3) Catechin (0.5 g) was mixed with 0.5 g of HMDA (70% solution in water) and 0.15 g of a 33 wt % aqueous solution of NaOH. Three samples were prepared with the proportions above. After that, they were reacted in an oven at 65, 100, and 185 °C overnight, respectively.
(4) Mimosa tannin (2 g) was mixed with 2 g of hexamethylenediamine (HMDA) (70% solution in water). Three samples were prepared with the proportions above, and they were reacted in an oven at 65, 100, and 185 °C overnight, respectively.
(5) Mimosa tannin (2 g) was mixed with 2 g of HMDA (70% solution in water) and 0.6 g of a 65 wt % aqueous solution *p*-toluenesulfonic acid (pTSA). Three samples were prepared with the proportions above. Then, they were reacted in an oven at 65, 100, and 185 °C overnight, respectively.
(6) Mimosa tannin (2 g) was mixed with 2 g of HMDA (70% solution in water) and 0.6 g of a 33 wt % aqueous solution NaOH. Again, three samples were prepared with the proportions above, and they were reacted in an oven at 65, 100, and 185 °C overnight, respectively.

The samples were mixed with a spatula because they become a paste after the addition of catechin or mimosa tannin. After the reaction in the oven, the samples prepared at 100 and 185 °C become a dry solid, while the samples prepared at 65 °C remained like a paste.

In the case of the mimosa tannin samples at 185 °C, they have not been analysed by MALDI because the spectra were not good enough due to the difficulty of their solubility in the acetone–water solution for their MALDI-ToF analysis.

### 2.2. Matrix-Assisted Laser Desorption Ionisation Time-of-Flight (MALDI-ToF) Mass Spectrometry Analysis

The spectra were recorded on a KRATOS Kompact MALDI AXIMA TOF 2 instrument (KRATOS Ana lytical, Shimadzu Europe Ltd., Manchester, UK). The irradiation source was a pulsed nitrogen laser with a wavelength of 337 nm. The time period of a laser pulse was 3 ns. The measurements were carried out using the following conditions: polarity = positive, flight path = linear, mass = high (20 kV acceleration voltage), 100–150 pulses per spectrum. The delayed extraction technique was used by applying delay times of 200–800 ns.

### 2.3. CP-MAS ^13^C NMR

Solid-state CP-MAS (cross-polarisation/magic-angle spinning) ^13^C NMR spectra of the aforementioned oven-dried solids were recorded on a Brüker MSL 300 spectrometer (Brüker France, Wissembourg, France) at a frequency of 75.47 MHz. Chemical shifts were calculated relative to tetramethyl silane (TMS). The rotor was spun at 4 kHz on a double-bearing 7 mm Bruker probe. The spectra were acquired with 5 s recycle delays, a 90° pulse of 5 s and a contact time of 1 ms. The number of transients was 3000.

The ^13^C NMR spectra were simulated with ACD/I-Lab version 12.0 (Advanced Chemistry Development Inc., Strasbourg, France) and with a free program online nmrdb.org [15] (https://www.nmrdb.org/).

## 3. Results and Discussion

### 3.1. Reactions of Catechin with Hexamethylene Diamine

#### 3.1.1. MALDI-ToF

While some of the peaks obtained by MALDI-ToF in the products obtained by reactions at 185 °C are the same as those in the cases at 100 °C, a greater number of different types of compounds are observed in the reactions at 100 °C. For the MALDI-ToF analysis of the reactions of catechin as a model compound, the spectra of the NaOH-catalysed reaction will be discussed, as the peaks are practically the same for the acid-catalysed and uncatalysed cases, the main differences being their relevant proportions. Two types of reactions appear to occur from the calculation of the MALDI masses found, namely (i) the formation of secondary amines by reaction of the hexamethylene diamine on the –OH groups of the flavonoid units; and (ii) the formation of –O^− +^NH_3_-salts between the amino group and some of the phenolic –OH groups of the tannin flavonoids.

**Figure 2:**
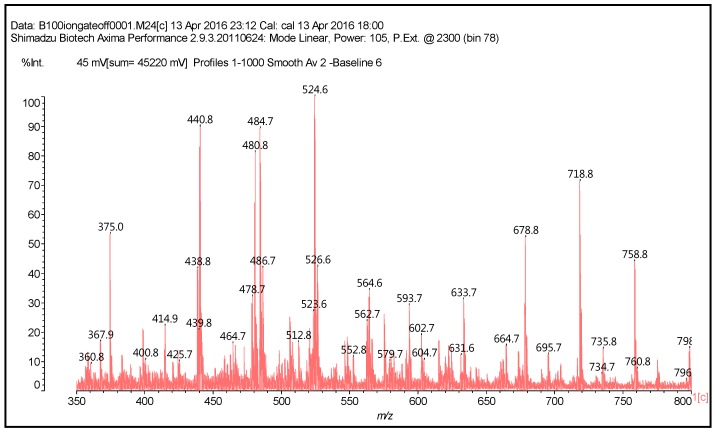
MALDI-ToF spectrum of the reaction of catechin with hexamethylene diamine at 185 °C, NaOH-catalysed. Range 350–800 Da.

**Figure 3:**
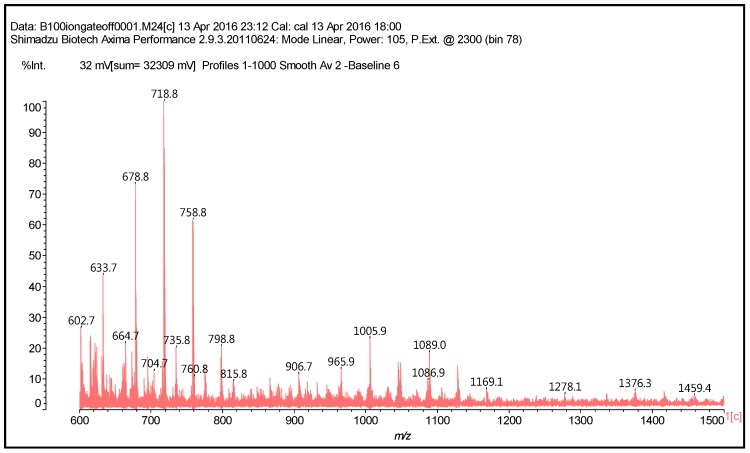
MALDI-ToF spectrum of the reaction of catechin with hexamethylene diamine at 185 °C, NaOH-catalysed. Range 600–1500 Da.

Thus, in the spectra obtained, the main peaks observed are reported in Table 1, and Figure 2 and Figure 3. There appears to be a clear period of 40 Da. This is a diamine with 2Na^+^ (not an unusual occurrence), thus 116 + 23 + 23 − 2 = 160 Da, giving the 160/4 = 40 Da period. The series of peaks that appears is then 798-758-718-678-634-594-553(small)-513 Da. From this series, for example, the repetition of peaks follows a 160 Da period such as 513 + 160 = 673 Da (678 Da), 594 + 160 = 754 Da (758 Da) (this is salt 4 × 117 = 755 Da), and 638 + 160 = 798 Da = 755 + 2Na^+^ = 801 − 2H^+^ = 799 Da.

**Figure 4:**
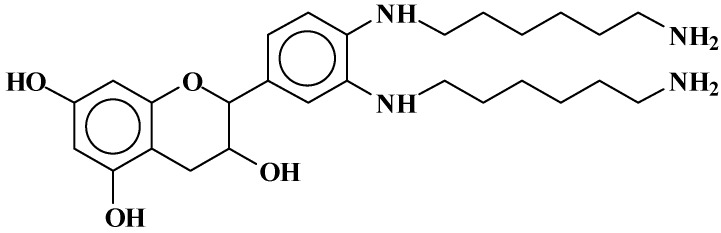
Example of covalent bonds structure at 509–512 Da.

The structures of the type of compounds more characteristic that formed (see Table 1) are thus as follows. At 509–512 Da (Figure 4), where the bonds formed are covalent.

**Figure 5:**
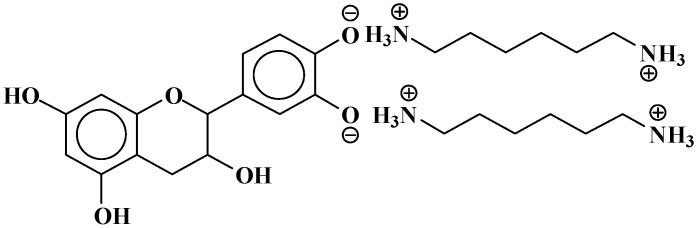
Example of ionic bonds salt structure at 524.6 Da.

At 524.6 Da (Figure 5), where the bonds formed are strongly ionic, thus forming a salt.

**Figure 6:**
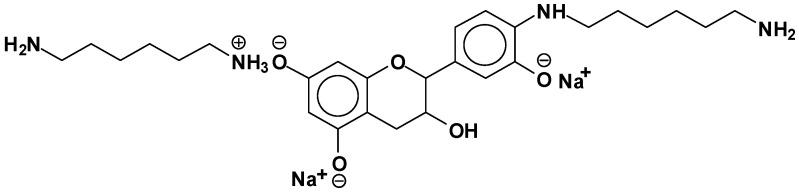
Example of mixed ionic and covalent bonds structure at 548 Da.

Additionally, mixed-bond species, such as the oligomer at 548 Da (Figure 6):

It must be pointed out that the structure shown above is the most likely, rather than the Na^+^ being attached to the N of the amine. This is so because, in general, a strong base is needed to abstract a proton from an amine. The NaOH used in the catalysis of the reaction is not strong enough for this. Furthermore, such sodium amides are strong bases themselves, which are not likely to coexist in the presence of protic compounds such as phenols. It is then most likely that the 548 Da peak belongs to a molecule that is the sodium salt of the phenolate ion. This is equally valid for structures such as the 564 Da peak observed for the mimosa tannin and other structures where Na^+^ is present. (Table 2).

**Figure 7:**
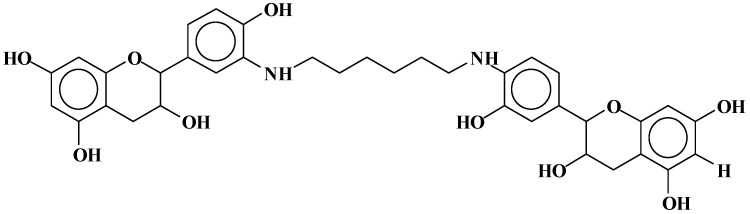
Example of covalent bonds dimer structure at 664 Da.

**Figure 8:**
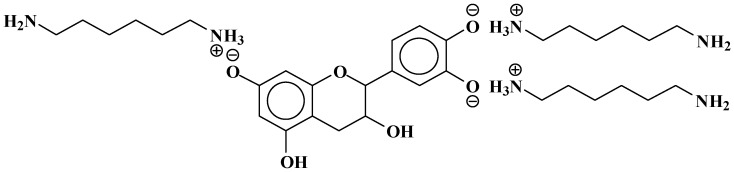
Example of ionic bonds salt structure at 661 Da.

**Figure 9:**
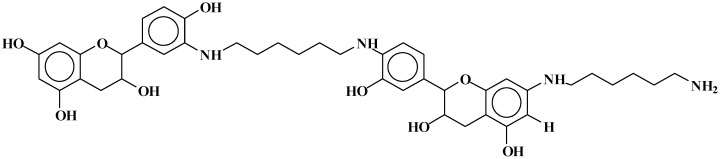
Example of covalent bonds dimer at 758 Da.

**Figure 10:**
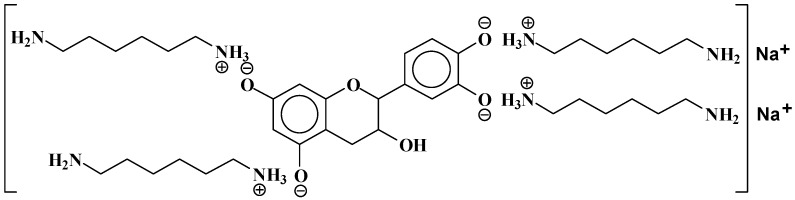
Example of ionic bonds salt structure at 798 Da.

**Figure 11:**
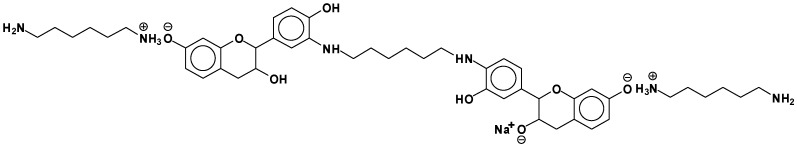
Example of covalent bonds dimer mixed with ionic salt bonds at 880 Da.

**Figure 12:**
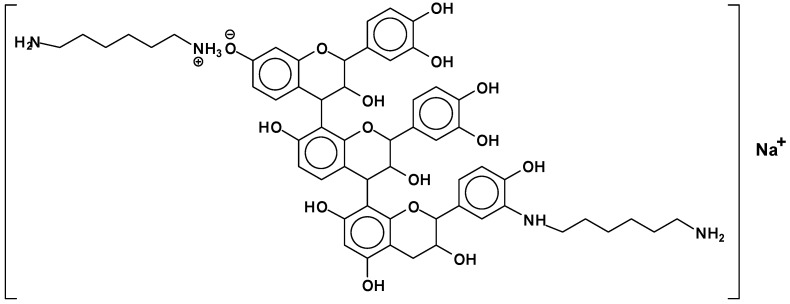
Example of a flavonoid trimer structure with amine reacted with mixed covalent bonds and ionic salt bonds at 1070 Da.

Dimers of two catechin monomers linked covalently through an hexamethylenediamine occurs, such as the peak occurring at 664 Da (Figure 7):

This can, however, also be interpreted as 638 + 1 × Na^+^ = 661 Da, thus an ionic salt such as (Figure 8):

Equally, at 758 Da (Figure 9):

At 798 Da (Figure 10):

At 880 Da (Figure 11):

At 1070 Da (Figure 12):

Higher oligomers in which catechin has dimerised also occurs, this being a fairly common reaction [16,17]. In these, the catechin dimer is linked covalently to either a catechin monomer or another catechin dimer, such as those shown by the peaks for the oligomers at 1169 Da = 1145 + 1 × Na^+^ (Figure 2) and at 1404 and 1459 Da, deprotonated.

**Figure 13:**
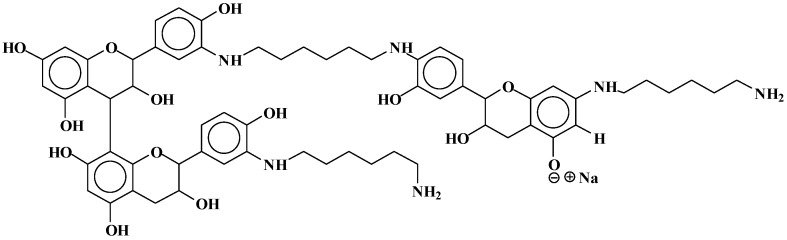
Example of flavonoid dimer and monomer covalently bridged by a diamine with other covalently linked diamines at 1169 Da.

**Figure 14:**
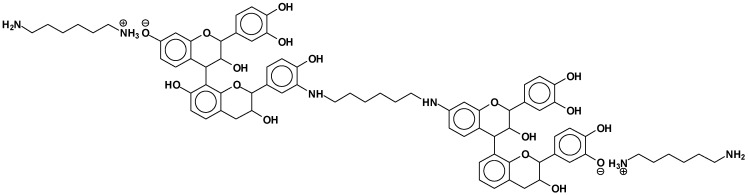
Example of two flavonoid dimers covalently bridged by a diamine with other ionic salt bonds linked diamines at 1404 Da.

**Figure 15:**
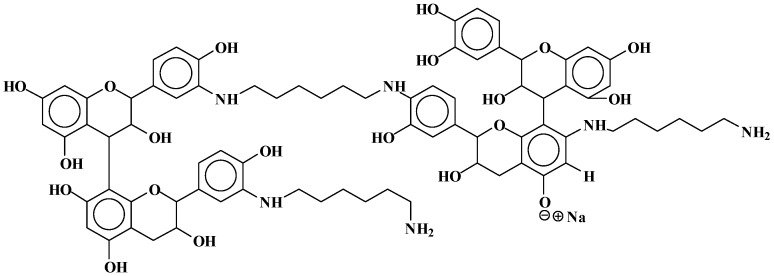
Example of two flavonoid dimers covalently bridged by a diamine with other covalently linked diamines at 1459 Da.

At 1169 Da (Figure 13):

At 1404 Da (Figure 14):

At 1459 Da, deprotonated (Figure 15): 

It must be made clear that the structures above are not the only possible isomers deduced from the peaks of the MALDI-ToF spectra, but that other isomer possibilities do exist for them. The existence of different isomers becomes clearer and is also confirmed later by the CP-MAS ^13^C NMR analysis.

#### 3.1.2. CP-MAS ^13^C NMR

In regard to the NMR spectra: the reactions occurring appear to be more advanced when the temperature is higher, while the reaction appears almost not to occur at the lower temperature of 65 °C. For this reason, the case of the reaction of catechin as a model compound with hexamethylene diamine catalysed by pTSA at 185 °C will be discussed first. The corresponding CP-MAS ^13^C NMR spectrum is shown in Figure 4.

**Figure 16:**
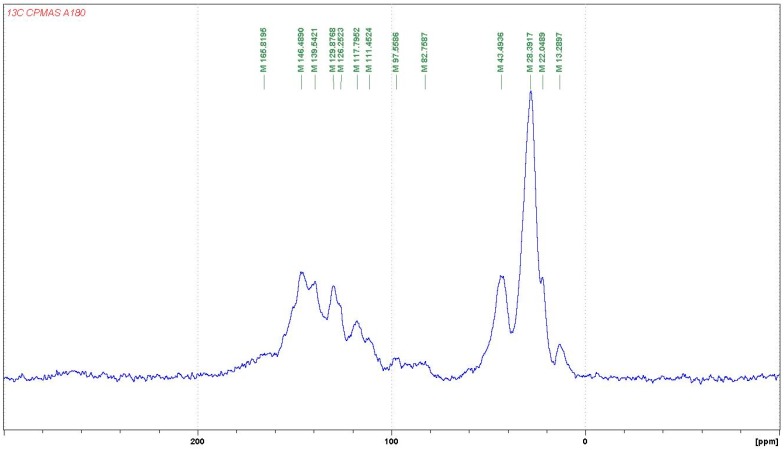
Cross-polarisation/magic-angle spinning (CP-MAS) ^13^C NMR spectrum of the reaction of catechin with hexamethylene diamine at 185 °C, *p*-toluenesulfonic acid (pTSA)-catalysed.

In Figure 16, first of all, the aliphatic carbon in position alpha to an –NH of the diamine reacted covalently with the tannin must have a shift of 43–44 ppm, while the same for an aliphatic amine not reacted should have a calculated shift of 41–42 ppm. Looking at the spectra of the 185 °C reactions, either pTSA- and NaOH-catalysed or uncatalysed (one reported in Figure 4, the others reported in the Supplementary Material), it can be noticed that the shift is at 42.9 ppm (uncatalysed), 43.2 ppm (NaOH-catalysed), and 43.5 ppm (pTSA-catalysed) indicating that at 185 °C, the amine has reacted covalently. This is confirmed by other indications. The shift for the C in β of the covalently reacted diamine should be at 30 ppm, while the unreacted one should be at 33–34 ppm. This peak is not visible at all in uncatalysed and pTSA-catalysed, as it is covered totally by the huge peak at 27–28 ppm, but appears as a slight shoulder at 33 ppm for the NaOH-catalysed case. It must be clearly pointed out that the shift of the top of the 43–44 ppm wide peak clearly changes when comparing the CP-MAS ^13^C NMR spectra of the three cases when the reaction is carried out at 100 °C (all spectra reported in the Supplementary Material). Thus, they are respectively at 41.9 ppm (pTSA-catalysed), 42.6 ppm (NaOH-catalysed), and 42.3 ppm. This indicates that, just based on NMR evidence, the type of bonds obtained in the reaction at 100 °C appears to be more uncertain, or at least that ionic bonds and covalent bonds are in different proportions according to the presence of different catalysts, or their absence. This implies that at the higher temperature of 185 °C, the reaction shift more towards the formation of covalently bound amines, the most found in pTSA catalysis, followed by NaOH catalysis, and least in the uncatalysed case.

**Figure 17:**
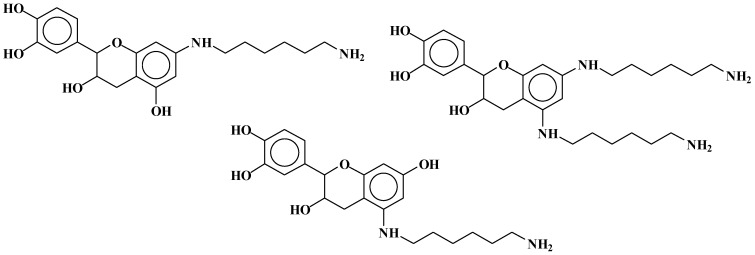
Covalently linked catechin A-ring-diamine structures observed by ^13^C NMR.

The other clear indication of the existence of the formation of covalent bonds between the amine and the catechin –OH groups is the considerable decrease of the peak at 155–157 ppm, indicating that the carbons C5 and C7 carrying the –OH groups on the A-ring have markedly decreased as they have reacted. This species formed by this reaction is defined by the appearance of a new peak at 139.5 ppm. This peak belongs to a flavonoid C5 and a C7 that have reacted covalently with an amine, indicating that the interpretation given to the MALDI spectra has been incomplete because there has been considerable reaction on the A-ring to form the following types of linkages (Figure 17).

**Figure 18:**
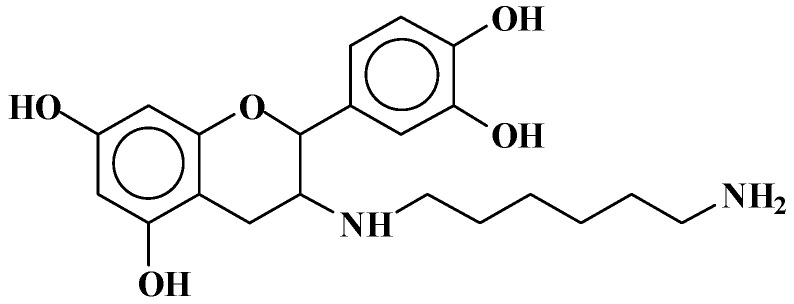
Covalently linked diamine onto C3 site of catechin.

This is not all. The total disappearance in the 180 °C spectra of the catechin C3 peak at 68–72 ppm indicates that even the alcoholic –OH on the C3 site has reacted covalently with the amine to form linkages of the type (Figure 18):

This being a rather unexpected occurrence.

The first question to be asked is then: are covalent bonds with the amine formed also with the carbons of the B-ring, as interpreted from the oligomers representation shown in the interpretation of the MALDI spectra?

**Figure 19:**
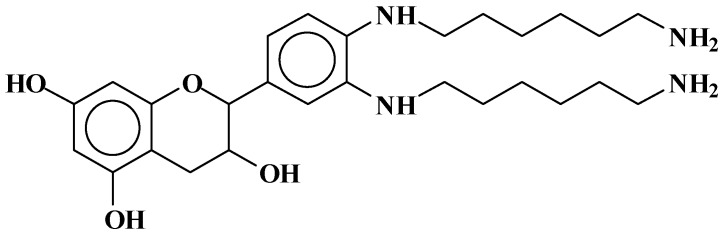
Covalently linked diamine onto catechin B-ring sites.

The answer to this question is clearly yes, as the covalent bonds are also formed at 185 °C on the B-ring as the 145–146 ppm peak belonging to the aromatic B-ring carbons carrying the phenolic –OH groups is also markedly smaller in all the catalysed and uncatalysed spectra at 185 °C. Moreover, the covalent bond formed transmit at 134–135 ppm, indicating that the 138–139 ppm peak belongs to both the covalently reacted A- and B-rings of catechin. Thus, linkages such as Figure 19 also occur.

**Figure 20:**
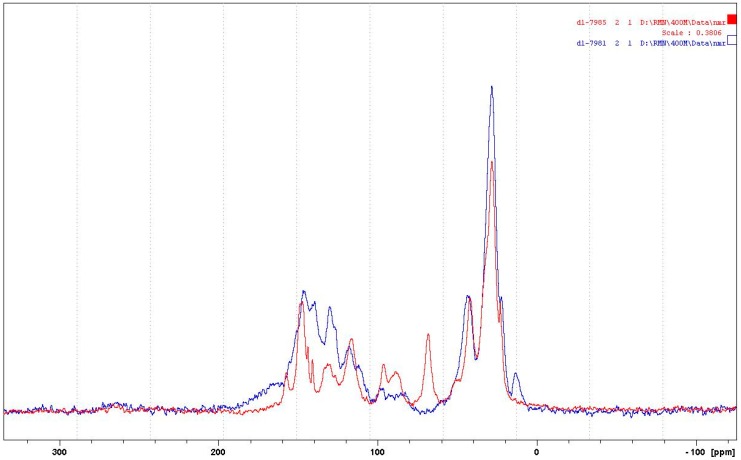
Superposition for comparison of the CP-MAS ^13^C NMR spectra of the reactions of catechin with hexamethylene diamine at 185 °C (blue curve) and at 100 °C (red curve), both pTSA-catalysed.

The simulation of the spectra with ACD/I-Lab yields a value of 41.8 ppm for the reacted amine, and 38.7 ppm for the unreacted amine. The online software at nmrdb.org [15] yields 44.0 ppm for the covalently reacted amine formed and 41.9 ppm for the nonreacted amine. The superposition of the pTSA-catalysed spectra at 185 and 100 °C shown in Figure 20 indicates that the formation of the amine is indeed occurring.

Furthermore, on the pTSA-catalysed 185 °C spectrum, a rotation band at 13 ppm occurs. If the rate of rotation that has caused it is subtracted, a well-defined peak at 133.8 ppm is obtained. This peak is hidden by other species. This chemical shift can also be simulated with an amination of the –OH group on the C3 of catechin and corresponds to the to the shift of the carbon in C1′. Moreover, the disappearing of the peak at 68 ppm observed on the pTSA-catalysed 100 °C spectrum and absent on the pTSA-catalysed 185 °C spectrum shows that the C3 carbon of the catechin has lost its –OH group, substituted with an –NH group (calculated at 67.6 ppm for catechin and 68 ppm for the 100 °C case, and at 55.8 ppm for the amination on C3 corresponding to the large peak at 55–59 ppm for the 180 °C case).

The second question is: do the ionic-type salt bonds, apparent in the MALDI spectra, really occur?

**Figure 21:**
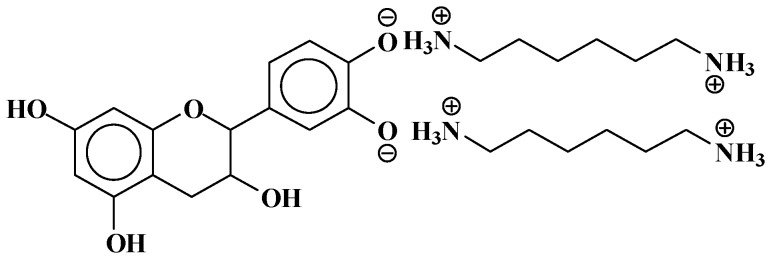
Example of structure of diamines linked to catechin B-ring sites by ionic salt bonds.

The response is also clearly positive. The huge peak at 27–29 ppm belongs to either diamine not reacted or to diamine linked as a totally ionised salt to structures of the type which follows. Thus, in both strongly acid- and strongly alkaline-catalysed reactions at 185 °C, it is certain that the rest of the amine is coordinated to catechin with linkages such as Figure 21, where the salts are formed with the –OHs of both the B- and A-rings of the catechin. It must be considered that unreacted diamine might be mixed with this, although the MALDI clearly indicates that the ionic bonds do exist. The presence of the 33 ppm shoulder in the spectrum of the alkali-catalysed 185 °C reaction product indicates that, for the amine, β carbons confirm that ionic bonds in quantity do occur.

In the case of the uncatalysed reaction, the amine should also be present in the same manner, thus partly ionically linked to the catechin or unreacted.

The last question to be answered by the NMR analysis is: what happens at lower temperature, namely at 100 °C?

The spectra are not reported here (they are available in the Supplementary Material), but the reaction is clearly less advanced, as should be expected. First of all, the C3 of the catechin has not reacted at all as the alcoholic C3–OH site shift exists and is big. Second, there is a clear covalent reaction of the amine on the catechin A-ring and some (lesser) reaction on the B-ring in the case of the pTSA-catalysed 100 °C spectrum. The reaction on the A-ring is also clear for the NaOH-catalysed 100 °C spectrum and also, but to a lesser extent, for the uncatalysed 100 °C spectrum. For these latter two, it does not appear that reaction on the B-ring does occur, or at least its proportion is minimal. Furthermore, no reactions on C3 appear to have occurred.

### 3.2. Reaction of Mimosa Tannin Extract with Hexamethylene Diamine

The same reactions were repeated by using mimosa tannin extract instead of the catechin model compound. The MALDI-ToF spectra of the reaction alkaline catalysis are shown in Figure 6 and Figure 7. The oligomer species obtained under alkaline catalysis are listed in Table 2. The MALDI spectra for the acid-catalysed and uncatalysed reactions at 100 °C and 65 °C are reported in the Supplementary Material.

**Figure 22:**
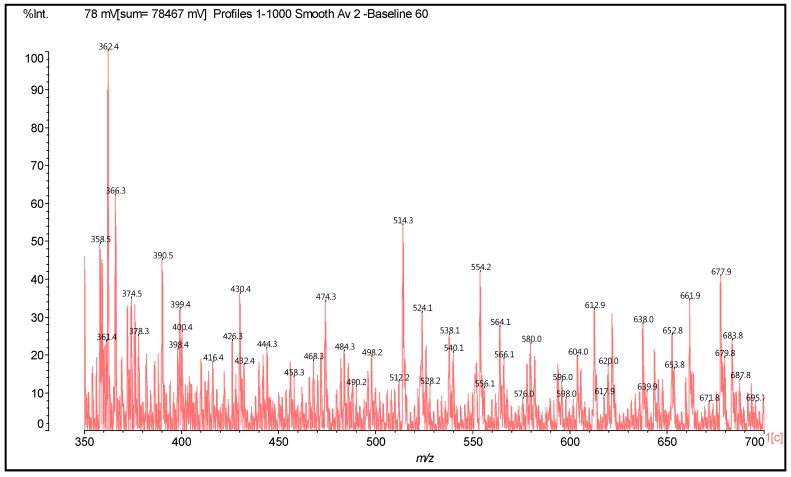
MALDI-ToF spectrum of the reaction of mimosa tannin extract with hexamethylene diamine at 100 °C, pTSA-catalysed. Range 350–700 Da.

**Figure 23:**
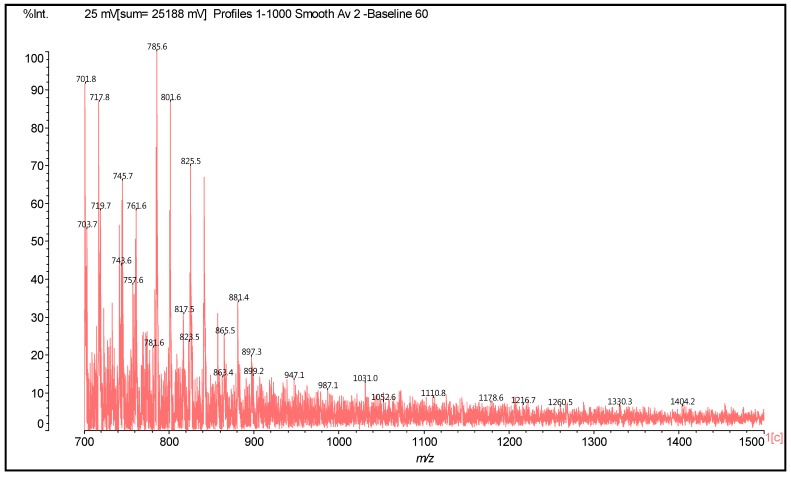
MALDI-ToF spectrum of the reaction of mimosa tannin extract with hexamethylene diamine at 100 °C, pTSA-catalysed. Range 700–1500 Da.

From Figure 22 and Figure 23, the same period of 40 Da is observed as in the case of catechin due to a diamine with 2 × Na^+^, thus 116 + 23 + 23 – 2 = 160 Da, thus 160/4 = 40 Da period: 801-761-719-677-638-596-554-514-474-430-390-350, is observed. The same type of reactions observed for the case of the catechin model compound appears to occur. Thus, both (1) substitution of the flavonoid units’ hydroxyl groups with the amino group of the amine and (2) the formation of –O^−^ Na^+^ salts appear to occur. Compounds in which one, two, and even three diamines are linked covalently to a flavonoid monomer unit are observed as, for example, the peaks at 374, 524, and 621 Da (Table 2). Equally, species in which one or more HMDA molecules are linked covalently to a flavonoid dimer occur as, for example, the ones represented by the peaks at 743 and 758 Da (Table 2), as well as species in which HMDA constitutes a bridge between two species such as two flavonoid dimers as, for example, the compounds at peaks 1260 and 1330 Da (Table 2). Conversely, species in which the amine is not covalently linked to a flavonoid unit, but rather a salt has been formed, are also present, such as, for example, the species at 390, 428–430, 661 Da, and many others as indicated in Table 2. Moreover, mixed species in which some HMDA molecules are linked covalently and some are linked by a salt bond to the same flavonoid are also present, as, for example, the species at peaks 638, 677, 761, and 1404 Da. Unreacted flavonoid oligomers—such as those represented by the peaks at 612, 881, 1178 Da, and others—are also present (Table 2).

The MALDI analysis of the catechin–HMDA reaction was done at 185 °C (and 100 and 65 °C in the Supplementary Material attached to this article), but was done at 100 °C for the mimosa tannin HMDA reaction for reasons of solubility. The catechin–HMDA sample at 185 °C, while solid, shows still more than sufficient solubility in acetone for analysis by MALDI. This is, however, not the case for the mimosa tannin–HMDA reaction that, at 185 °C, has hardened and is practically insoluble. This is why the MALDI was done on the 100 °C tannin/HMDA reaction: it was much less polymerised and the majority of it was still soluble in acetone. Equally, it was the reason why the NMR of the solids of the catechin-hardened material was done. It must be pointed out that the number average degree of polymerisation (DPn) of mimosa tannin is around 4.5, thus 4–5 flavonoid units linked together. This is why a hardened tridimensional network is reached more rapidly in the reaction of HMDA with the tannin than with a catechin monomer.

## 4. Conclusions

A novel reaction of amines with flavonoid tannin is described. The reaction of hexamethylenediamine with catechin, a flavonoid monomer used as a model compound of condensed tannins, and with mimosa tannin were studied. The reactions were carried out at three different temperatures: 65, 100, and 185 °C. The reaction products obtained were analysed by MALDI-TOF and CP-MAS ^13^C NMR, and the structures of the chemical species formed were indicated. Two reactions occurred under both alkaline and acid conditions, namely (i) the reaction of the amine with the phenolic hydroxy groups of the tannin, leading to polycondensation resins; and (ii) a reaction leading to the formation of ionic bonds between the protonated amino groups of the amine and the hydroxyl groups of the flavonoid structure for both the catechin and the tannin. Hardened, insoluble resins were formed for the tannin at the higher temperature, and resins insoluble in water but soluble in acetone were formed at 100 °C.

- At 185 °C, there are covalent bonds between the amine and the A- and B-rings of the catechin and also with the aliphatic C3 site of the catechin. The A-ring appears to react first, before the B-rings.
- At 185 °C, there is a high proportion of ionic bonds between the amine and the A- and B-rings of the catechin, while presence of some unreacted amine cannot be excluded.
- At 100 °C, the proportion of ionic bonds appears to predominate.
- At 100 °C, there are covalent bonds between the amine and the A- and B-rings of the catechin for the pTSA-catalysed reaction (reaction A), with the A-rings having reacted more.
- At 100 °C, there are covalent bonds between the amine and the A-rings of the catechin for the NaOH-catalysed reaction, but less or even no reaction on the B-ring. There are even fewer covalent bonds in the uncatalysed reaction.
- At 180 °C, and to a lesser extent also at 100 °C, mimosa tannin reacting with hexamethylene diamine forms hard, condensed solids, be it uncatalysed or alkali- or acid-catalysed.
- At 180 °C, the condensation solid formed is hardly soluble in acetone water.
- The reaction of mimosa tannin or similar condensed tannins with a diamine is fast.
- In regard to the influence of different catalysts, their influence is minimal other than to accelerate the reaction. To this purpose, reactions at three different temperatures (but without any catalysts) were done with similar results, and the analysis results are shown in the Supplementary Material.

## Figures and Tables

**Table 1:** MALDI-ToF peaks interpretation. NaOH-catalysed reaction of catechin and hexamethylenediamine at 100 °C *.

289.9 Da = Catechin alone
509–512 Da = catechin-(hexamethylenediamine)_2_ (509 Da calculated)
524.6 Da = catechin-(hexamethylenediamine)_2_ ionic salt
526 Da = 524 diprotonated
552.8 Da = catechin-(hexamethylenediamine)_2_ + 3 × Na^+^ (calculated 553 Da)
579.7 Da = catechin-(hexamethylenediamine)_2_ + 4 × Na^+^ (calculated 582 Da)
602.7 Da = catechin dimer + Na^+^ (calculated 601 Da)
605 Da = catechin-(hexamethylenediamine)_3_ + Na^+^
631.6–633.7 Da = catechin-(hexamethylenediamine)_3_ + 2 × Na^+^ (calculated 630 Da)
664 Da = catechin-hexamethylenediamine-catechin (660 Da calculated)
However, also:
638 + 1 × Na^+^ = catechin-(hexamethylenediamine)_3_ ionic salt (calculated 661 Da)
678 Da = catechin-(hexamethylenediamine)_3_ + 4 × Na^+^ (calculated 675 Da)
758 Da = catechin-hexamethylenediamine-catechin-hexamethylenediamine
798 Da = catechin-(hexamethylenediamine)_4_ + 2 × Na^+^, ionic salt.
1169 Da = hexamethylenediamine-catechindimer-hexamethylenediamine-catechin-hexamethylenediamine + 1 × Na^+^
1459 Da = hexamethylenediamine-catechindimer-hexamethylenediamine-catechindimer-hexamethylenediamine, diprotonated

* In the spectra, there is a clear period of 40 Da. This is a diamine with 2Na^+^, thus 116 + 23+ 23 − 2 = 160 Da, thus with 2 × Na^+^, not an unusual occurrence. Also, 160/4 = 40 Da period, thus a series 798-758-718-678-634-594-553(small)-513 Da Thus, 513 + 160 = 673 Da (678 Da); thus 594 + 160 = 754 Da (758 Da). This is a salt 4 × 117 = 755 Da; thus 638 + 160 = 798 Da = 755 Da + 2Na^+^ = 801 Da − 2H^+^ = 799 Da.

**Table 2:** Oligomer species formed during the reaction at 100 °C between mimosa tannin extract and hexamethylene diamine in the NaOH-catalysed reaction.

Experimental	Calculated (Da)	Description of Calculation (Da)	Description	Number Ionic Bonds	Number Covalent Bonds
372–374	372	274 + 116 − 18	F-HMDA	-	1
390	390	274 + 116	F(−)(+)HMDA	1	-
410	410	290 − 17 + 114 + 23	F-HMDA(−)(+)Na	-	1
426	426	306 + 114 − 17 + 23	G-HMDA(−)(+)Na	-	1
428–430	428	290 + 115 + 23	C(−)(+)HMDA(−)(+)Na	1	-
444	444	306 + 115 + 23	G(−)(+)HMDA(−)(+)Na	1	-
488	488	274 − 17 + 115 + 116	HMDA(+)(−)F-HMDA	1	1
468	468	274 + 114 × 2 − 17 × 2	(HMDA-F-HMDA) less 2H^+^	-	2
484	484	290 + 114 × 2 − 17 × 2	(HMDA-C-HMDA) less 2H^+^	-	2
500	500	304 + 2 + 114 × 2 − 17 − 17	(HMDA-G-HMDA) less 2H+	-	2
524	524	306 + 114 + 115 – 17 − 17 + 23	(G[HMDA]_2_)(−)(+)Na	-	2
525	525	290 + 114 + 115 − 17 + 23	HMDA-C(−)(+)HMDA(−)(+)Na	1	1
528	528	274 + 116 + 114 + 23	HMDA(+)(−)F(−)(+)HMDA(−)(+)Na	2	-
540	542	306 + 115 × 2 − 17 + 23	[HMDA(+)(−)G-HMDA](−)(+)Na	1	1
562	562	272 + 288 + 2	Dimer	-	-
564	564	306 + 114 + 115 − 17 + 23 × 2	Na(+)(−)HMDA(+)(−)G-HMDA(−)(+)Na	1	1
578	578	288 + 288 + 2	Dimer	-	-
612	610	304 + 304 + 2	Dimer	-	-
617	618	306 + 115 × 2 − 17 × 2 + 116	HMDA(+)(−)G(HMDA)_2_	1	2
621	622	306 + 115 × 2+ 114 − 17 × 3 + 23	[G(HMDA)_3_](−)(+)Na	-	3
638	640	306 + 115 × 3 − 17 − 17 + 23	[HMDA(+)(−)G(HMDA)_2_](−)(+)Na	1	2
643	644	272 + 272 + 2 + 114 − 17	F-F-HMDA	-	1
642	642	272 + 288 + 2 + 114 − 17 × 2	F-HMDA-C	-	2
653	654	306 + 116 × 3	G[(−)(+)HMDA]_3_	3	-
661	660	272 + 288 + 2 + 115 − 17	F-C-HMDA	-	1
661	662	272 + 272 + 2 + 116	F-F(−)(+)HMDA	1	-
677	676	306 + 116 × 2+ 115 + 23	[HMDA(+)(−)]_2_ G (−)(+)HMDA(−)(+)Na	3	-
677	678	272 + 288 + 2 + 116	F-C(−)(+)HMDA	1	-
682	682	272 + 288 + 2 + 114 − 17 + 23	[F-C-HMDA]	-	1
687	688	272 + 2 + 115 × 3 + 114 − 17 × 4 + 23	[F(HMDA)_4_](−)(+)Na	-	4
695	694	288 + 288 + 2 + 116	C-C(−)(+)HMDA	1	-
701	701	272 + 288 + 2 + 116 + 23	[F-C(−)(+)HMDA](−)(+)Na	1	-
716	717	288 + 288 + 2 + 116 + 23	[C-C(−)(+)HMDA](−)(+)Na	1	-
723	723	272 + 272 + 2 + 114 + 115 − 17 × 3	F-HMDA-F-HMDA	-	3
745	744	274 + 274 + 114 + 116 − 17 × 2	F-HMDA-F(−)(+)HMDA	-	-
740–743	742	274 + 274 + 2 + 115 × 2 − 17 × 2	HMDA-F-F-HMDA	-	2
757	758	272 + 288 + 2 + 115 × 2 − 17 × 2	HMDA-F-C-HMDA	-	2
761	760	274 + 290 + 114 − 17 × 2 + 116	F-HMDA-C(−)(+)HMDA	1	2
857	857	272 + 272 + 288 + 2 + 23	Trimer	-	-
865	866	272 + 288 + 304 + 2	Trimer	-	-
881	880	272 × 2 + 2 + 115 × 3 − 17 × 2 + 23	[(HMDA(+)(−))_2_ (F-HMDA-F)](−)(+)Na	2	2
881	882	288 × 2 + 304 + 2	Trimer	-	-
898	898	272 × 2 + 2+ 114 × 3 − 17 + 23	[(HMDA(+)(−))_2_ F-F-HMDA](−)(+)Na	2	1
898	898	288 + 304 × 2 + 2	Trimer	-	-
906	905	288 × 2 + 304 + 2 + 23	Trimer	-	-
921	921	288 + 304 × 2 + 2 + 23	Trimer	-	-
1052	1052	857 + 114 + 115 − 17 × 2	[(F-C-F)(HMDA)_2_](−)(+)Na	-	2
1069	1070	857 + 115 × 2 − 17	[HMDA-(F-C-F)(−)(+)HMDA](−)(+)Na	1	1
1178	1178	288 × 4 + 2 + 23	Tetramer	-	-
1260	1258	288 × 2 + 2 + 116 − 18 × 2 + 288 × 2 + 2 + 23 − 1	[C-C-HMDA-C-C](−)(+)Na	-	2
1330	1334	288 × 2 + 2 + 116 × 2 − 18 × 3 + 288 × 2 + 2	C-C-HMDA-C-C-HMDA	-	3
1404	1404	272 × 2 + 2 + 116 × 3 − 18 × 2 + 272 × 2 + 2	HMDA(+)(−)F-F-HMDA-F-F(−)(+)HMDA	2	2

F = fisetinidin; C = catechin or robinetinidin; G = gallocatechin; HMDA = hexamethylenediamine; “-” = covalent bond; “(+)(−)” = ionic bond; “(+)(−)Na = Na^+^ linked to flavonoid units phenolic –OHs as –O^−^ Na^+^.

## Supplementary Materials

The following are available online at www.mdpi.com/2073-4360/9/2/37/s1, Table S1: Structures determined by MALDI ToF of the reaction of mimosa + hexamethylenediamine + NaOH at 100 °C, Table S2: Structures determined by MALDI ToF of the reaction of mimosa +hexamethylenediamine + pTSA at 100 °C, Table S3: Structures determined by MALDI ToF of the uncatalysed reaction of mimosa + hexamethylenediamine at 100 °C, Table S4: Structures determined by MALDI ToF of the reaction of mimosa + hexamethylenediamine + NaOH at 65 °C, Table S5: Structures determined by MALDI ToF of the reaction of mimosa + hexamethylenediamine + pTSA at 65 °C, Table S6: Structures determined by MALDI ToF of the uncatalysed reaction of mimosa + hexamethylenediamine at 65 °C, Table S7: Structures determined by MALDI ToF of the uncatalysed reaction of catechin monomer + hexamethylenediamine at 185 °C, Table S8: Structures determined by MALDI ToF of the uncatalysed reaction of catechin monomer + hexamethylenediamine at 100 °C, Table S9: Structures determined by MALDI ToF of the uncatalysed reaction of catechin monomer + hexamethylenediamine at 65 °C, Figure S1: MALDI ToF spectrum of the reaction of mimosa + hexamethylenediamine + NaOH at 100 °C. 350–700 Da range, Figure S2: MALDI ToF spectrum of the reaction of mimosa + hexamethylenediamine + NaOH at 100 °C. 700–1500 Da range, Figure S3: MALDI ToF spectrum of the reaction of mimosa + hexamethylenediamine + pTSA at 100 °C. 700–2200 Da range, Figure S4: MALDI ToF spectrum of the uncatalysed reaction of mimosa + hexamethylenediamine at 100 °C. 350–700 Da range, Figure S5: MALDI ToF spectrum of the uncatalysed reaction of mimosa + hexamethylenediamine at 100 °C. 700–1500 Da range. Figure S6: MALDI ToF spectrum of the reaction of mimosa + hexamethylenediamine + NaOH at 65 °C. 350–700 Da range, Figure S7: MALDI ToF spectrum of the reaction of mimosa + hexamethylenediamine + NaOH at 65 °C. 700–1500 Da range, Figure S8: MALDI ToF spectrum of the reaction of mimosa + hexamethylenediamine + pTSA at 65 °C 350–700 Da range, Figure S9: MALDI ToF spectrum of the reaction of mimosa + hexamethylenediamine + pTSA at 65 °C 700–1500 Da range, Figure S10: MALDI ToF spectrum of the uncatalysed reaction of mimosa + hexamethylenediamine at 65 °C. 350–700 Da range, Figure S11: MALDI ToF spectrum of the uncatalysed reaction of mimosa + hexamethylenediamine at 65 °C. 700–1500 Da range, Figure S12: MALDI ToF of the uncatalysed reaction of catechin monomer + hexamethylenediamine at 185 °C. 330–600 Da range, Figure S13: MALDI ToF of the uncatalysed reaction of catechin monomer + hexamethylenediamine at 185 °C. 350–2000 Da range, Figure S14: MALDI ToF spectrum of the uncatalysed reaction of catechin monomer + hexamethylenediamine at 100 °C. 350–800 Da range, Figure S15: MALDI ToF spectrum of the uncatalysed reaction of catechin monomer + hexamethylenediamine at 100 °C. 600–2000 Da range, Figure S16: MALDI ToF spectrum of the uncatalysed reaction of catechin monomer + hexamethylenediamine at 65 °C. 330–600 Da range, Figure S17: MALDI ToF spectrum of the uncatalysed reaction of catechin monomer + hexamethylenediamine at 65 °C. 600–1500 Da range, Figure S18: MALDI ToF spectrum of the reaction of catechin monomer + hexamethylenediamine + NaOH at 185 °C. 350–800 Da range, Figure S19: MALDI ToF spectrum of the reaction of catechin monomer + hexamethylenediamine + NaOH at 185 °C. 600–1500 Da range, Figure S20: MALDI ToF spectrum of the reaction of catechin monomer + hexamethylenediamine + NaOH at 100 °C. 350–800 Da range, Figure S21: MALDI ToF spectrum of the reaction of catechin monomer + hexamethylenediamine + NaOH at 100 °C. 600–1500 Da range, Figure S22: MALDI ToF spectrum of the reaction of catechin monomer + hexamethylenediamine + NaOH at 65 °C. 350–800 Da range, Figure S23: MALDI ToF spectrum of the reaction of catechin monomer + hexamethylenediamine + NaOH at 65 °C. 600–1500 Da range, Figure S24: MALDI ToF spectrum of the reaction of catechin monomer + hexamethylenediamine + pTSA at 185 °C. 350–800 Da range, Figure S25: MALDI ToF spectrum of the reaction of catechin monomer + hexamethylenediamine + pTSA at 100 °C. 350–800 Da range, Figure S26: MALDI ToF spectrum of the reaction of catechin monomer + hexamethylenediamine + pTSA at 100 °C. 600–1500 Da range, Figure S27: MALDI ToF spectrum of the reaction of catechin monomer + hexamethylenediamine + pTSA at 65 °C. 350–800 Da range, Figure S28: MALDI ToF spectrum of the reaction of catechin monomer + hexamethylenediamine + pTSA at 65 °C. 400–900 Da range, Figure S29: CP MAS ^13^C NMR spectrum of the reaction of catechin monomer + hexamethylenediamine + pTSA at 185 °C, Figure S30: CP MAS ^13^C NMR spectrum of the reaction of catechin monomer + hexamethylenediamine + pTSA at 100 °C, Figure S31: CP MAS ^13^C NMR spectrum of the reaction of catechin monomer + hexamethylenediamine + NaOH at 185 °C, Figure S32: CP MAS ^13^C NMR spectrum of the reaction of catechin monomer + hexamethylenediamine + NaOH at 100 °C, Figure S33: CP MAS ^13^C NMR spectrum of the uncatalysed reaction of catechin monomer + hexamethylenediamin at 185 °C, Figure S34: CP MAS ^13^C NMR spectrum of the uncatalysed reaction of catechin monomer + hexamethylenediamin at 100 °C. Thus, (1) MALDI-ToF spectra and tables of reaction products of the uncatalysed, NaOH-catalysed, and pTSA-catalysed reactions of mimosa tannin extract with hexamethylene diamine at 100 and 65 °C. (2) MALDI-ToF spectra and tables of reaction products of the uncatalysed reactions of catechin with hexamethylene diamine at 185, 100, and 65 °C.

## References

[1] Pizzi A., Pizzi A. (1983). Tannin-based wood adhesives. Wood Adhesives Chemistry and Technology.

[2] Pizzi A. (1994). Advanced Wood Adhesives Technology.

[3] Garcia R., Pizzi A. (1998). Polycondensation and autocondensation networks in polyflavonoid tannins. I. Final networks. J. Appl. Polym. Sci..

[4] Garcia R., Pizzi A. (1998). Polycondensation and autocondensation networks in polyflavonoid tannins. II. Polycondensation vs. Autocondensation. J. Appl. Polym. Sci..

[5] Masson E., Merlin A., Pizzi A. (1996). Comparative kinetics of the induced radical autocondensation of polyflavonoid tannins. I. Modified and non-modified tannins. J. Appl. Polym. Sci..

[6] Masson E., Pizzi A., Merlin A. (1996). Comparative kinetics of the induced radical autocondensation of polyflavonoid tannins. III. Micellar reactions vs. cellulose surface catalysis. J. Appl. Polym. Sci..

[7] Masson E., Pizzi A., Merlin A. (1997). Comparative kinetics of: The induced radical autocondensation of polyflavonoid tannins. II. Flavonoid units effects. J. Appl. Polym. Sci..

[8] Itoh M., Hattori H., Tanabe K. (1974). The acidic properties of TiO_2_-SiO_2_ and its catalytic activities for the amination of phenol, the hydration of ethylene and the isomerization of butane. J. Catal..

[9] Iranpoor N., Panahi F. (2014). Direct Nickel-catalyzed amination of phenols via C-O Bond activation using 2,4,6-Trichloro-1,3,5-triazine (TCT) as reagent. Adv. Synth. Catal..

[10] Yu J., Wang Y., Zhang P., Wu J. (2013). Direct amination of phenols under metal-free conditions. Synlett.

[11] Kim H.J., Kim J., Cho S.H., Chang S. (2011). Intermolecular oxidative C-N bond formation under metal-free conditions: Control of chemoselectivity between aryl sp2 and benzylic sp3 C-H Bond imidation. J. Am. Chem. Soc..

[12] Hashida K., Makino R., Ohara S. (2009). Amination of pyrogallol nucleus of condensed tannins and related polyphenols by ammonia water treatment. Holzforschung.

[13] Kida K., Suzuki M., Takagaki A., Nanjo F. (2002). Deodorizing effects of tea catechins on amines and ammonia. Biosci. Biotechnol. Biochem..

[14] Braghiroli F., Fierro V., Pizzi A., Rode K., Radke W., Delmotte L., Parmentier J., Celzard A. (2013). Condensation reactions of flavonoid tannins with ammonia. Ind. Crop. Prod..

[15] Binev Y., Marques M.M., Aires-de-Sousa J. (2007). Prediction of 1H NMR coupling constants with associative neural networks trained for chemical shifts. J. Chem. Inf. Model..

[16] Ohara S., Hemingway R.W. (1991). Condensed tannins: The formation of a diarylpropanol-catechinic acid dimer from base-catalyzed reactions of (+)-catechin. J. Wood Chem. Technol..

[17] Hashida K., Ohara S., Makino R. (2003). Base-catalyzed reactions of (−)-epicatechin: Formation of enantiomers of base-catalyzed reaction products from (+)-catechin. J. Wood Chem. Technol..

