# Supplementary material for: Polycondensation Resins by Flavonoid Tannins Reaction with Amines
Source: Polymers (Basel). 2017 Jan 25;9(2):37. doi: 10.3390/polym9020037 (PMC6432223; doi:10.3390/polym9020037)
Supplement: Supplementary file 1 [file polymers-09-00037-s001.pdf]

Supplementary Materials: Polycondensation Resins by Flavonoid Tannins Reaction with Amines

Francisco-Jose Santiago-Medina, Antonio Pizzi, Maria Cecilia Basso, Luc Delmotte and Alain Celzard

1. Mimosa Tannin Reactions

For each sample the number refers to the temperature (65, 100 and 185 °C) and the letter sAB = mimosa + hexamethylenediamine A = mimosa + hexamethylenediamine + pTSA and B = mimosa + hexamethylenediamine + NaOH.

In the case of the samples at 180 °C, they have not been analysed because the spectra are not very good due to these samples unable to be well-dissolved in the acetone–water solution before theirs measurements in the MALDI.

AB is mimosa + hexamethylenediamine without no acid or base catalyst

Legend

F = fisitinidin

C = catechin

G = gallocatechin

HMDA= Hexamethylenediamine

“-” = covalent bond

“(+)(−)” = ionic bond

“(+)(−)Na = Na^+ linked to flavonoid units phenolic –OHs as $–O^-Na^+$

B100

Table S1. Structures determined by MALDI ToF of the reaction of mimosa + hexamethylenediamine + NaOH at 100 °C.

Experimental	Calculated (Da)	Descrip. Cal (Da)	Description	Number Ionic Bonds	Number Covalent Bonds
372	372	274 + 116 − 18	F-HMDA	-	1
390	390	274 + 116	F(−)(+)HMDA	1	-
410	410	290 − 17 + 114 + 23	F-HMDA(−)(+)Na	-	1
426	426	306 + 114 − 17 + 23	G-HMDA(−)(+)Na	-	1
428	428	290 + 115 + 23	C(−)(+)HMDA(−)(+)Na	1	-
444	444	306 + 115 + 23	G(−)(+)HMDA(−)(+)Na	1	-
488	488	274 − 17 + 115 + 116	HMDA(+)(−)F-HMDA	1	1
468	468	274 + 114 × 2 − 17 × 2	(HMDA-F-HMDA) less 2H+	-	2
484	484	290 + 114 × 2 − 17 × 2	(HMDA-C-HMDA) less 2H+	-	2
500	500	304 + 2 + 114 × 2 − 17 − 17	(HMDA-G-HMDA) less 2H+	-	2
524	524	306 + 114 + 115 − 17 − 17+ 23	(G[HMDA]$_2$)(−)(+)Na	-	2
525	525	290 + 114 + 115 − 17 + 23	HMDA-C(−)(+)HMDA(−)(+)Na	1	1
528	528	274 + 116 + 114 + 23	HMDA(+)(−)F(−)(+)HMDA(−)(+)Na	2	-
540	542	306 + 115 × 2 − 17 + 23	[HMDA(+)(−)G-HMDA](−)(+)Na	1	1
562	562	272 + 288 + 2	Dimer	-	-
564	564	306 + 114 + 115 − 17 + 23 × 2	Na(+)(−)HMDA(+)(−)G-HMDA(−)(+)Na	1	1
578	578	288 + 288 + 2	Dimer	-	-
612	610	304 + 304 + 2	Dimer	-	-
617	618	306 + 115 × 2 − 17 × 2 + 116	HMDA(+)(−)G(HMDA)$_2$	1	2
621	622	306 + 115 × 2 + 114 − 17 × 3 + 23	[G(HMDA)$_3$](−)(+)Na	-	3
638	640	306 + 115 × 3 − 17 − 17 + 23	[HMDA(+)(−)G(HMDA)$_2$](−)(+)Na	1	2
643	644	272 + 272 + 2 + 114 − 17	F-F-HMDA	-	1

642	642	272 + 288 + 2 + 114 − 17 × 2	F-HMDA-C	-	2
653	654	306 + 116 × 3	G[(−)(+)HMDA]$_3$	3	-
661	660	272 + 288 + 2 + 115 − 17	F-C-HMDA	-	1
661	662	272 + 272 + 2 + 116	F-F(−)(+)HMDA	1	-
677	676	306 + 116 × 2 + 115 + 23	[HMDA(+)(−)]$_2$ G (−)(+)HMDA(−)(+)Na	3	-
677	678	272 + 288 + 2 + 116	F-C(−)(+)HMDA	1	-
682	682	272 + 288 + 2 + 114 − 17 + 23	[F-C-HMDA]	-	1
687	688	272 + 2 + 115 × 3 + 114 ×17 × 4 + 23	[F(HMDA)$_4$](−)(+)Na	-	4
695	694	288 + 288 + 2 + 116	C-C(−)(+)HMDA	1	-
701	701	272 + 288 + 2 + 116 + 23	[F-C(−)(+)HMDA](−)(+)Na	1	-
716	717	288 + 288 + 2 + 116 + 23	[C-C(−)(+)HMDA](−)(+)Na	1	-
723	723	272 + 272 + 2 + 114 + 115 − 17 × 3	F-HMDA-F-HMDA	-	3
745	744	274 + 274 + 114 + 116 − 17 × 2	F-HMDA-F(−)(+)HMDA	-	-
740-743	742	274 + 274 + 2 + 115 × 2 − 17 × 2	HMDA-F-F-HMDA	-	2
757	758	272 + 288 + 2 + 115 × 2 − 17 × 2	HMDA-F-C-HMDA	-	2
761	760	274 + 290 + 114 − 17 × 2 + 116	F-HMDA-C(−)(+)HMDA	1	2
857	857	272 + 272 + 288 + 2 + 23	Trimer	-	-
865	866	272 + 288 + 304 + 2	Trimer	-	-
881	880	272 × 2 + 2 + 115 × 3 − 17 × 2 + 23	[(HMDA(+)(−))$_2$ (F-HMDA-F)](−)(+)Na	2	2
881	882	288 × 2 + 304 + 2	Trimer	-	-
898	898	272 × 2 + 2 + 114 × 3 − 17 + 23	[(HMDA(+)(−))$_2$ F-F-HMDA](−)(+)Na	2	1
898	898	288 + 304 × 2 + 2	Trimer	-	-
906	905	288 × 2 + 304 + 2 + 23	Trimer	-	-
921	921	288 + 304 × 2 + 2 + 23	Trimer	-	-
1052	1052	857 + 114 + 115 − 17 × 2	[(F-C-F)(HMDA)$_2$](−)(+)Na	-	2
1069	1070	857 + 115 × 2 − 17	[HMDA-(F-C-F)(−)(+)HMDA](−)(+)Na	1	1
1178	1178	288 × 4 + 2 + 23	Tetramer	-	-
1260	1258	288 × 2 + 2 + 116 − 18 × 2+288 × 2+2+23-1	[C-C-HMDA-C-C](−)(+)Na	-	2
1330	1334	288x2+2+116 × 2-18 × 3 + 288 × 2 + 2	C-C-HMDA-C-C-HMDA	-	3
1404	1404	272 × 2 + 2 + 116 × 3 − 18 × 2 + 272 × 2 + 2	HMDA(+)(−)F-F-HMDA-F-F(−)(+)HMDA	2	2

Period of 40 Da due to a diamine with $2Na^+$, thus 116 + 23 + 23 − 2 = 160 Da, thus 160/4 = 40 Da period: 801-61-719-677-638-596-554-514-474-430-390-350.

B100

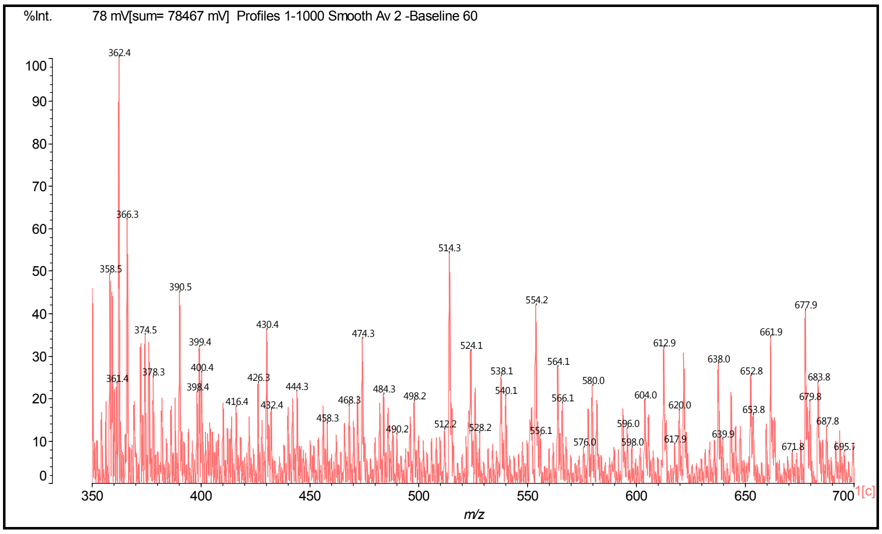

Figure S1. MALDI ToF spectrum of the reaction of mimosa + hexamethylenediamine + NaOH at 100 °C; 350–700 Da range.

B100

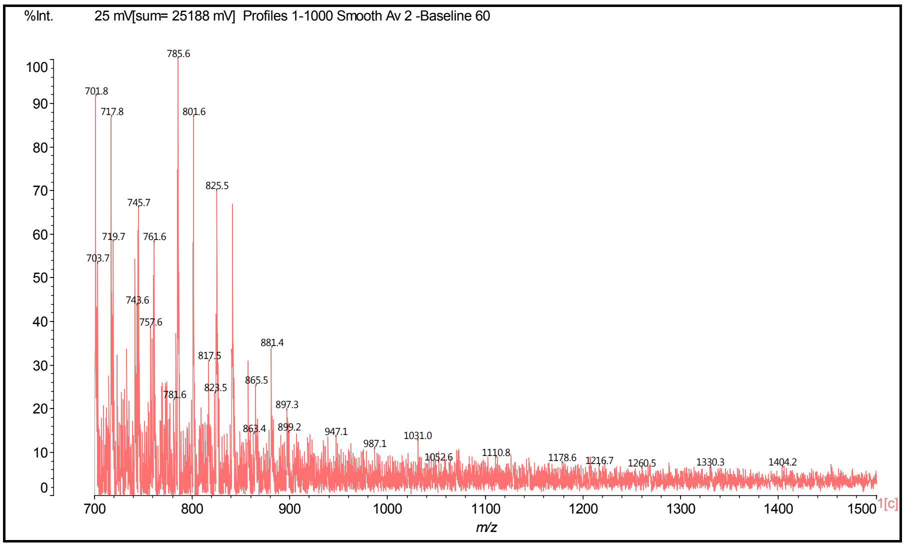

Figure S2. MALDI ToF spectrum of the reaction of mimosa + hexamethylenediamine + NaOH at 100°C; 700–1500 Da range.

A100

Table S2. Structures determined by MALDI ToF of the reaction of mimosa + hexamethylenediamine + pTSA at 100 °C.

Experimental	Calculated (Da)	Descrip. Cal (Da)	Description	Number Ionic Bonds	Number Covalent Bonds
371	372	274 + 116 − 18	F-HMDA	-	1
390	390	274 + 116	F(−)(+)HMDA	1	-
410	410	290 − 17 + 114 + 23	F-HMDA(−)(+)Na		1
412	412	274 + 115 + 23	F(−)(+)HMDA(−)(+)Na	1	
428	428	290 + 115 + 23	C(−)(+)HMDA(−)(+)Na	1	
444	444	306 + 115 + 23	G(−)(+)HMDA(−)(+)Na	1	
486 or 490	488	274 − 17 + 115 + 116	HMDA(+)(−)F-HMDA	1	1
486	484	290 + 114 × 2 − 17 × 2	(HMDA-C-HMDA) +2H+	-	2
524	524	306 + 114 + 115 − 17 − 17 + 23	(G[HMDA]$_2$)(−)(+)Na	-	2
526	525	290 + 114 + 115 − 17 + 23	HMDA-C(−)(+)HMDA(−)(+)Na	1	1
528	528	274 + 116 + 114 + 23	HMDA(+)(−)F(−)(+)HMDA(−)(+)Na	2	
540	542	306 + 115 × 2 − 17 + 23	[HMDA(+)(−)G-HMDA](−)(+)Na	1	1

564	564	306 + 114 + 115 − 17 + 23 × 2	Na(+)(−)HMDA(+)(−)G-HMDA(−)(+)Na	1	1
568	568	274 + 115 × 3 − 17 × 3	F[HMDA]$_3$	-	3
578	578	288 + 288 + 2	Dimer (small)	-	-
612	610	304 + 304 + 2	Dimer	-	-
621	622	306 + 115 × 2 + 114 − 17 × 3 + 23	[G(HMDA)$_3$](−)(+)Na	-	3
626	626	274 + 114 × 3 − 17 + 23	[HMDA-F((−)(+)HMDA)$_2$](−)(+)Na	2	1
640	640	306 + 115 × 3 − 17 − 17 + 23	[HMDA(+)(−)G(HMDA)$_2$](−)(+)Na	1	2
643	644	272 + 272 + 2 + 114 − 17	F-F-HMDA	-	1
643	642	272 + 288 + 2 + 114 − 17 × 2	F-HMDA-C	-	2
661	660	272 + 288 + 2 + 115 − 17	F-C-HMDA	-	1
661	662	272 + 272 + 2 + 116	F-F(−)(+)HMDA	1	-
677	676	306 + 116 × 2 + 115 + 23	[HMDA(+)(−)]$_2$ G (−)(+)HMDA(−)(+)Na	3	-
679	678	272 + 288 + 2 + 116	F-C(−)(+)HMDA	1	-
683	682	272 + 288 + 2 + 114 − 17 + 23	[F-C-HMDA]	-	1
695	694	288 + 288 + 2 + 116	C-C(−)(+)HMDA	1	-
700	701	272 + 288 + 2 + 116 + 23	[F-C(−)(+)HMDA](−)(+)Na	1	-
706	706	274 + 115 × 4 − 17 × 3 + 23	[(HMDA)$_3$F(−)(+)HMDA](−)(+)Na	1	3
717	717	288 + 288 + 2 + 116 + 23	[C-C(−)(+)HMDA](−)(+)Na	1	-
723	723	272 + 272 + 2 + 114 + 115 − 17 × 3	F-HMDA-F-HMDA	-	3
745	744	274 + 274 + 114 + 116 − 17 × 2	F-HMDA-F(−)(+)HMDA	-	-
758	758	272 + 288 + 2 + 115 × 2 − 17 × 2	HMDA-F-C-HMDA	-	2
761	760	274 + 290 + 114 − 17 × 2 + 116	F-HMDA-C(−)(+)HMDA	1	2
761	760	272 + 272 + 2 + 115 − 17 + 116	HMDA-F-F(−)(+)HMDA	1	1
777	776	272 + 288 + 2 + 114 × 2 − 17	HMDA-F-C(−)(+)HMDA	1	1
857	857	272 + 272 + 288 + 2 + 23	Trimer	-	-
880	880	272 − 2 + 2 + 115 × 3 − 17 × 2 + 23	[(HMDA(+)(−))$_2$ (F-HMDA-F)](−)(+)Na	2	2
882	882	288 × 2 + 304 + 2	Trimer	-	-
897	898	272 × 2 + 2 + 114 × 3 − 17 + 23	[(HMDA(+)(−))$_2$ F-F-HMDA](−)(+)Na	2	1
897	898	288 + 304 × 2 + 2	Trimer	-	-
906	905	288 × 2 + 304 + 2 + 23	Trimer	-	-
921	921	288 + 304 × 2 + 2 + 23	Trimer	-	-

Two period of around 44 and 43 Da, respectively, are clear. The first from 1260 Da until 1876 Da and the second one from 1849 Da until 2067 Da. Something similar is found in the spectrum of the samples prepared at 180 °C, which they could barely be dissolved. Thus, it can be quite likely to be a measurement error. A third period of 40 Da, due to a diamine with 2 Na^+ + 116 + 23 + 23 − 2 = 160/4, is observed from 801 Da until 350 Da.

A 100

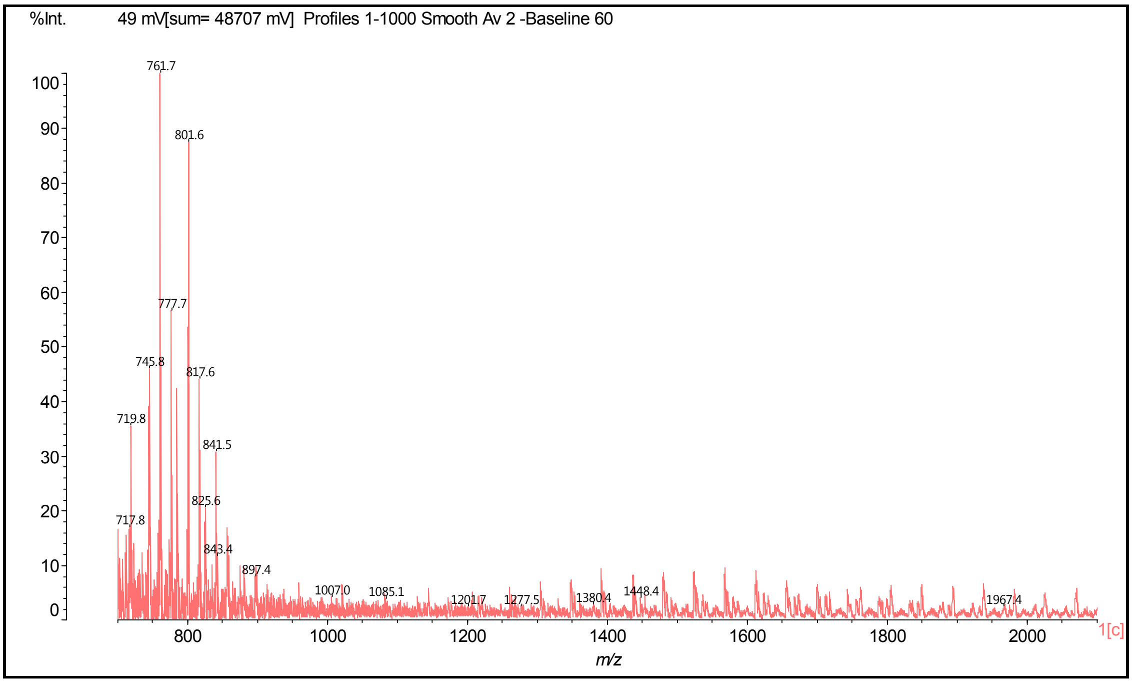

Figure S3. MALDI ToF spectrum of the reaction of mimosa + hexamethylenediamine + pTSA at 100°C; 700–2200 Da range.

sAB100

Table S3. Structures determined by MALDI ToF of the uncatalysed reaction of mimosa + hexamethylenediamine at 100°C.

Experimental	Calculated (Da)	Descrip. Cal (Da)	Description	Number Ionic Bonds	Number Covalent Bonds
371	372	274 + 116 − 18	F-HMDA	-	1
390	390	274 + 116	F(−)(+)HMDA	1	-
410	410	290-17 + 114 + 23	F-HMDA(−)(+)Na		1
444	444	306 + 115 + 23	G(−)(+)HMDA(−)(+)Na	1	
488	488	274 − 17 + 115 + 116	HMDA(+)(−)F-HMDA	1	1
468	468	274 + 114 × 2 − 17 × 2	(HMDA-F-HMDA) less 2H+	-	2
486	484	290 + 114 × 2 − 17 × 2	(HMDA-C-HMDA) +2H+	-	2
508	507	290 + 115 + 114 − 17 × 2 + 23	[C(HMDA)$_2$](+)(−)Na	-	2
524	524	306 + 114 + 115 − 17 − 17 + 23	(G[HMDA]$_2$)(−)(+)Na	-	2
526	525	290 + 114 + 115 − 17 + 23	HMDA-C(−)(+)HMDA(−)(+)Na	1	1
528	528	274 + 116 + 114 + 23	HMDA(+)(−)F(−)(+)HMDA(−)(+)Na	2	-
560	560	306 + 114 × 2 + 3 + 23	[G((−)(+)HMDA)$_2$](−)(+)Na	2	-
564	564	306 + 114 + 115 − 17 + 23 × 2	Na(+)(−)HMDA(+)(−)G-HMDA(−)(+)Na	1	1
567	568	274 + 115 × 3 − 17 × 3	F[HMDA]$_3$	-	3
579	578	288 + 288 + 2	Dimer	-	-
612	610	304 + 304 + 2	Dimer	-	-
619	618	306 + 115 × 2 − 17 × 2 + 116	HMDA(+)(−)G(HMDA)$_2$	1	2
622	622	306 + 115 × 2 + 114 − 17 × 3 + 23	[G(HMDA)$_3$](−)(+)Na	-	3
640	640	306 + 115 × 3 − 17 − 17 + 23	[HMDA(+)(−)G(HMDA)$_2$](−)(+)Na	1	2
643	644	272 + 272 + 2 + 114 − 17	F-F-HMDA	-	1
643	642	272 + 288 + 2 + 114 − 17 × 2	F-HMDA-C	-	2
661	660	272 + 288 + 2 + 115 − 17	F-C-HMDA	-	1
661	662	272 + 272 + 2 + 116	F-F(−)(+)HMDA	1	-
677	676	306 + 116 × 2 + 115 + 23	[HMDA(+)(−)]$_2$ G (−)(+)HMDA(−)(+)Na	3	-
679	678	272 + 288 + 2 + 116	F-C(−)(+)HMDA	1	-
683	682	272 + 288 + 2 + 114 − 17 + 23	[F-C-HMDA]	-	1
695	694	288 + 288 + 2 + 116	C-C(−)(+)HMDA	1	-
700	701	272 + 288 + 2 + 116 + 23	[F-C(−)(+)HMDA](−)(+)Na	1	-
719	717	288 + 288 + 2 + 116 + 23	[C-C(−)(+)HMDA](−)(+)Na	1	-
723	723	272 + 272 + 2 + 114 + 115 − 17 × 3	F-HMDA-F-HMDA	-	3
759	758	272 + 288 + 2 + 115 × 2 − 17 × 2	HMDA-F-C-HMDA	-	2
761	760	274 + 290 + 114 − 17 × 2 + 116	F-HMDA-C(−)(+)HMDA	1	2
761	760	272 + 272 + 2 + 115 − 17 + 116	HMDA-F-F(−)(+)HMDA	1	1
776	776	272 + 288 + 2 + 114 × 2 − 17	HMDA-F-C(−)(+)HMDA	1	1
857	857	272 + 272 + 288 + 2 + 23	Trimer	-	-
1069	1070	857 + 115 × 2 − 17	[HMDA-(F-C-F)(−)(+)HMDA](−)(+)Na	1	1

The same period of 40 Da from 801 until 350 is observed.

sAB100

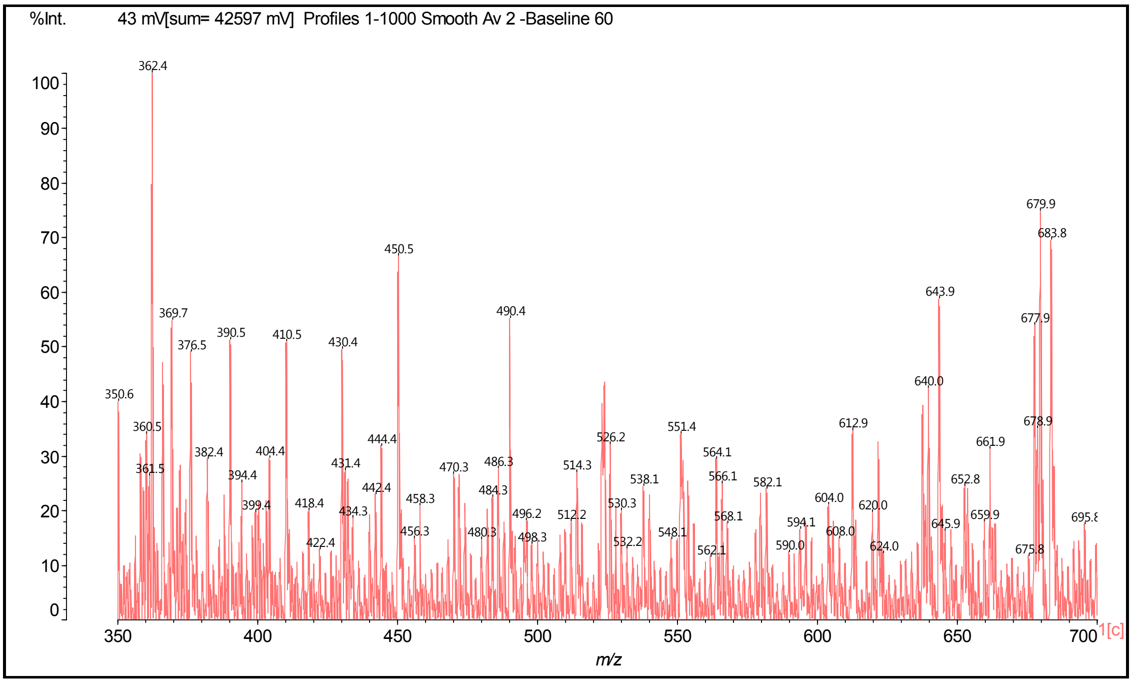

Figure S4. MALDI ToF spectrum of the uncatalysed reaction of mimosa + hexamethylenediamine at 100 °C; 350–700Da range.

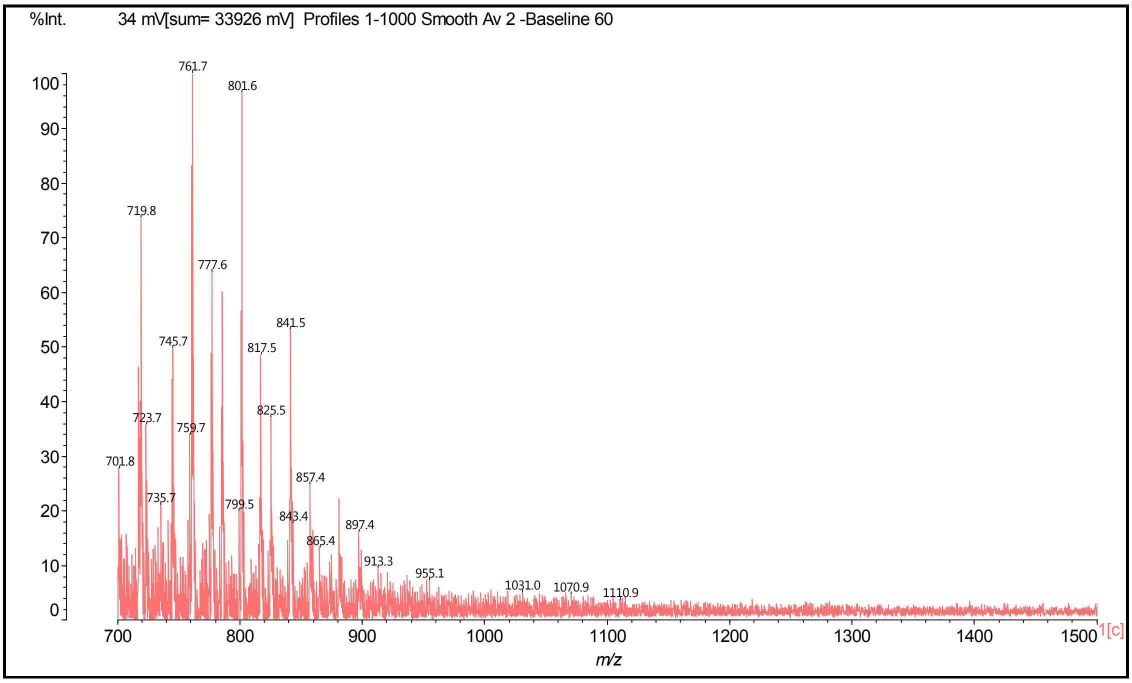

Figure S5. MALDI ToF spectrum of the uncatalysed reaction of mimosa + hexamethylenediamine at 100 °C; 700–1500 Da range.

In the three next samples, which were prepared at 65 °C, have also been found the same period of 40 Da, from 801 Da until 350 Da.

B65

Table S4. Structures determined by MALDI ToF of the reaction of mimosa + hexamethylenediamine + NaOH at 65 °C.

Experimental	Calculated (Da)	Descrip. Cal (Da)	Description	Number Ionic Bonds	Number Covalent Bonds
372	372	274 + 116 – 18	F-HMDA	-	1
390	390	274 + 116	F(−)(+)HMDA	1	-
445	444	306 + 115 + 23	G(−)(+)HMDA(−)(+)Na	1	
488	488	274 – 17 + 115 + 116	HMDA(+)(−)F-HMDA	1	1
466	468	274 + 114 × 2 – 17 × 2	(HMDA-F-HMDA) less 2H+	-	2
485	484	290 + 114 × 2 – 17 × 2	(HMDA-C-HMDA) +2H+	-	2
502	502	274 + 114 + 114	F[(−)(+)HMDA]$_2$ less 2H+	2	
507	507	290 + 115 + 114 – 17 × 2 + 23	[C(HMDA)$_2$](+)(−)Na	-	2
523	524	306 + 114 + 115 – 17 – 17 + 23	(G[HMDA]$_2$)(−)(+)Na	-	2
526	525	290 + 114 + 115 – 17 + 23	HMDA-C(−)(+)HMDA(−)(+)Na	1	1
542	542	306 + 115 × 2 – 17 + 23	[HMDA(+)(−)G-HMDA](−)(+)Na	1	1
563	562	272 + 288 + 2	Dimer	-	
563	564	306 + 114 + 115 – 17 + 23 × 2	Na(+)(−)HMDA(+)(−)G-HMDA(−)(+)Na	1	1
578	578	288 + 288 + 2	Dimer	-	-
612	610	304 + 304 + 2	Dimer	-	-
619	618	306 + 115 × 2 – 17 × 2 + 116	HMDA(+)(−)G(HMDA)$_2$	1	2
622	622	306 + 115 × 2 + 114 – 17 × 3 + 23	[G(HMDA)$_3$](−)(+)Na	-	3
643	644	272 + 114 × 3 + 2 + 23	-	3	-
643	644	272 + 272 + 2 + 114 – 17	F-F-HMDA	-	1
643	642	272 + 288 + 2 + 114 – 17 × 2	F-HMDA-C	-	2
652	654	306 + 116 × 3	G[(−)(+)HMDA]$_3$	3	-
661	660	272 + 288 + 2 + 115 – 17	F-C-HMDA	-	1
661	662	272 + 272 + 2 + 116	F-F(−)(+)HMDA	1	-
677	676	306 + 116 × 2 + 115 + 23	[HMDA(+)(−)]$_2$ G (−)(+)HMDA(−)(+)Na	3	-
678	678	272 + 288 + 2 + 116	F-C(−)(+)HMDA	1	-
683	682	272 + 288 + 2 + 114 – 17 + 23	[F-C-HMDA]	-	1
693	694	288 + 288 + 2 + 116	C-C(−)(+)HMDA	1	-
700	701	272 + 288 + 2 + 116 + 23	[F-C(−)(+)HMDA](−)(+)Na	1	-
719	717	288 + 288 + 2 + 116 + 23	[C-C(−)(+)HMDA](−)(+)Na	1	-
723	723	272 + 272 + 2 + 114 + 115 – 17 × 3	F-HMDA-F-HMDA	-	3
759	758	272 + 288 + 2 + 115 × 2 – 17 × 2	HMDA-F-C-HMDA	-	2
761	760	274 + 290 + 114 – 17 × 2 + 116	F-HMDA-C(−)(+)HMDA	1	2
761	760	272 + 272 + 2 + 115 – 17 + 116	HMDA-F-F(−)(+)HMDA	1	1
776	776	272 + 288 + 2 + 114 × 2 – 17	HMDA-F-C(−)(+)HMDA	1	1
857	857	272 + 272 + 288 + 2 + 23	Trimer	-	-
865	866	272 + 288 + 304 + 2	Trimer	-	-
908	905	288 × 2 + 304 + 2 + 23	Trimer	-	-

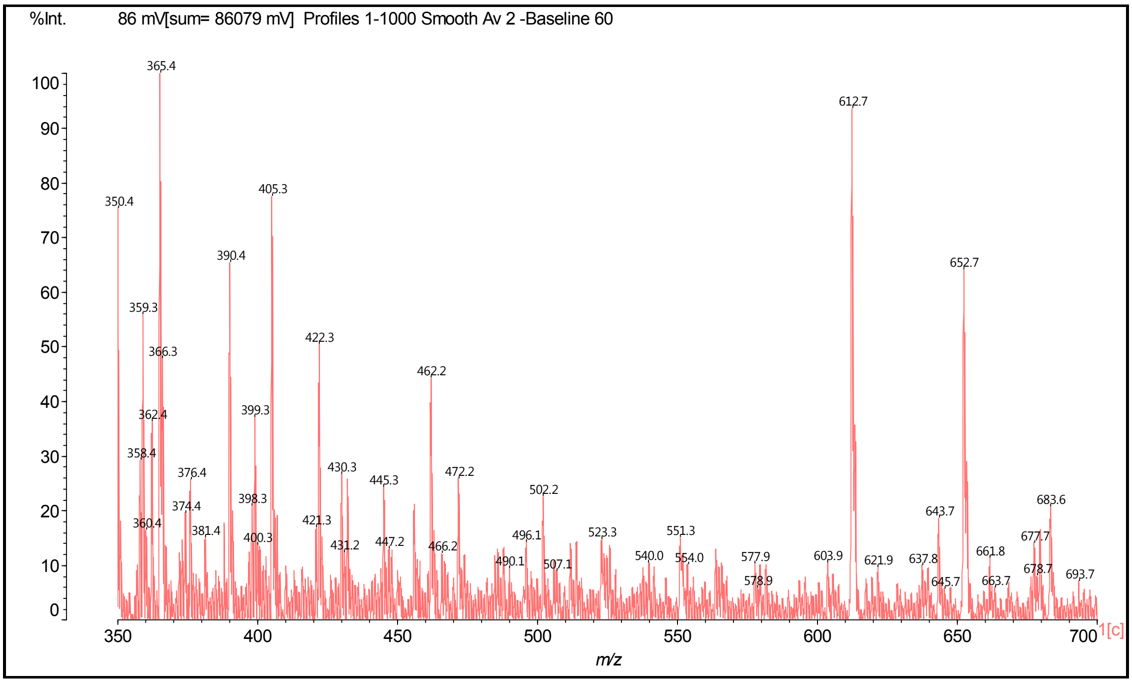

Figure S6. MALDI ToF spectrum of the reaction of mimosa + hexamethylenediamine + NaOH at 65 °C; 350–700 Da range.

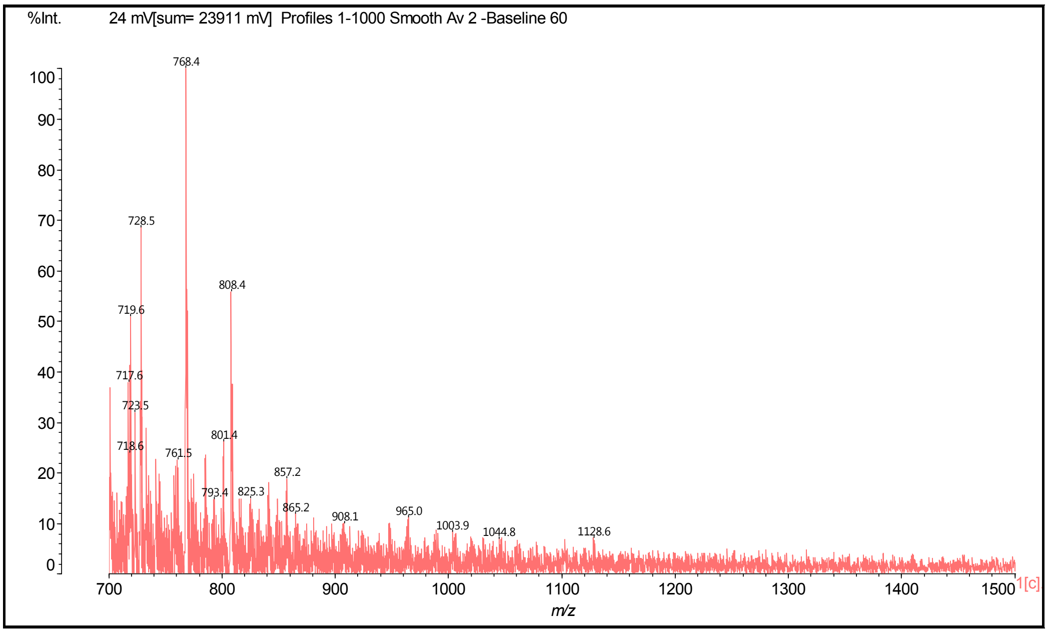

Figure S7. MALDI ToF spectrum of the reaction of mimosa + hexamethylenediamine + NaOH at 65 °C; 700–1500 Da range.

A65

Table S5. Structures determined by MALDI ToF of the reaction of mimosa + hexamethylenediamine + pTSA at 65 °C.

Experimental	Calculated (Da)	Descrip. Cal (Da)	Description	Number Ionic Bonds	Number Covalent Bonds
372	372	274 + 116 – 18	F-HMDA	-	1
390	390	274 + 116	F(−)(+)HMDA	1	-
410	410	290 – 17 + 114 + 23	F-HMDA(−)(+)Na		1
412	412	274 + 115 + 23	F(−)(+)HMDA(−)(+)Na	1	-
428	428	290 + 115 + 23	C(−)(+)HMDA(−)(+)Na	1	-

446	444	306 + 115 + 23	G(−)(+)HMDA(−)(+)Na	1	-
488	488	274 − 17 + 115 + 116	HMDA(+)(−)F-HMDA	1	1
486	486	290 + 115 × 2 − 17 × 2	(HMDA-C-HMDA)	-	2
501	500	304 + 2 + 114 × 2 − 17 − 17	(HMDA-G-HMDA) less 2H+	-	2
526	524	306 + 114 + 115 − 17 − 17 + 23	(G[HMDA]$_2$)(−)(+)Na	-	2
526	525	290 + 114 + 115 − 17 + 23	HMDA-C(−)(+)HMDA(−)(+)Na	1	1
540	542	306 + 115 × 2 − 17 + 23	[HMDA(+)(−)G-HMDA](−)(+)Na	1	1
568	568	274 + 115 × 3 − 17 − 17 − 17	F(HMDA)$_3$	-	3
612	610	304 + 304 + 2	Dimer	-	-
623	622	306 + 115 × 2 + 114 − 17 × 3 + 23	[G(HMDA)$_3$](−)(+)Na	-	3
639	640	306 + 115 × 3 − 17 − 17 + 23	[HMDA(+)(−)G(HMDA)$_2$](−)(+)Na	1	2
643	644	274 + 116 × 3 + 23 − 1	[F((−)(+)HMDA)$_3$](−)(+)Na	3	-
643	644	272 + 272 + 2 + 114 − 17	F-F-HMDA	-	1
642	642	272 + 288 + 2 + 114 − 17 × 2	F-HMDA-C	-	2
655	654	306 + 116 × 3	G[(−)(+)HMDA]$_3$	3	-
659	658	306 + 116 × 3 − 18 + 23 − 1	[HMDA-G((−)(+)HMDA)$_2$](−)(+)Na	2	1
659	660	272 + 288 + 2 + 115 − 17	F-C-HMDA	-	1
661	662	272 + 272 + 2 + 116	F-F(−)(+)HMDA	1	-
677	676	306 + 116 × 2 + 115 + 23	[HMDA(+)(−)]$_2$ G (−)(+)HMDA(−)(+)Na	3	-
679	678	272 + 288 + 2 + 116	F-C(−)(+)HMDA	1	-
682	682	272 + 288 + 2 + 114 − 17 + 23	[F-C-HMDA]	-	1
695	694	288 + 288 + 2 + 116	C-C(−)(+)HMDA	1	-
723	723	272 + 272 + 2 + 114 + 115 − 17 × 3	F-HMDA-F-HMDA	-	3
723	724	274 + 116 × 4 − 18 × 2 + 23 − 1	[(HMDA)$_2$F((−)(+)HMDA)$_2$](−)(+)Na	2	2
745	744	274 + 274 + 114 + 116 − 17 × 2	F-HMDA-F(−)(+)HMDA	-	-
740-743	742	274 + 274 + 2 + 115 × 2 − 17 × 2	HMDA-F-F-HMDA	-	2
759	758	272 + 288 + 2 + 115 × 2 − 17 × 2	HMDA-F-C-HMDA	-	2
761	760	274 + 290 + 114 − 17 × 2 + 116	F-HMDA-C(−)(+)HMDA	1	2
761	760	272 + 272 + 2 + 115 − 17 + 116	HMDA-F-F(−)(+)HMDA	1	1
777	776	272 + 288 + 2 + 115 − 17 + 116	HMDA-F-C(−)(+)HMDA	1	1
857	857	272 + 272 + 288 + 2 + 23	Trimer	-	-
865	866	272 + 288 + 304 + 2	Trimer	-	-
897	898	272 × 2 + 2 + 114 × 3 − 17 + 23	[(HMDA(+)(−))$_2$ F-F-HMDA](−)(+)Na	2	1
897	898	288 + 304 × 2 + 2	Trimer	-	-
906	905	288 × 2 + 304 + 2 + 23	Trimer	-	-
925	921	288 + 304 × 2 + 2 + 23	Trimer	-	-

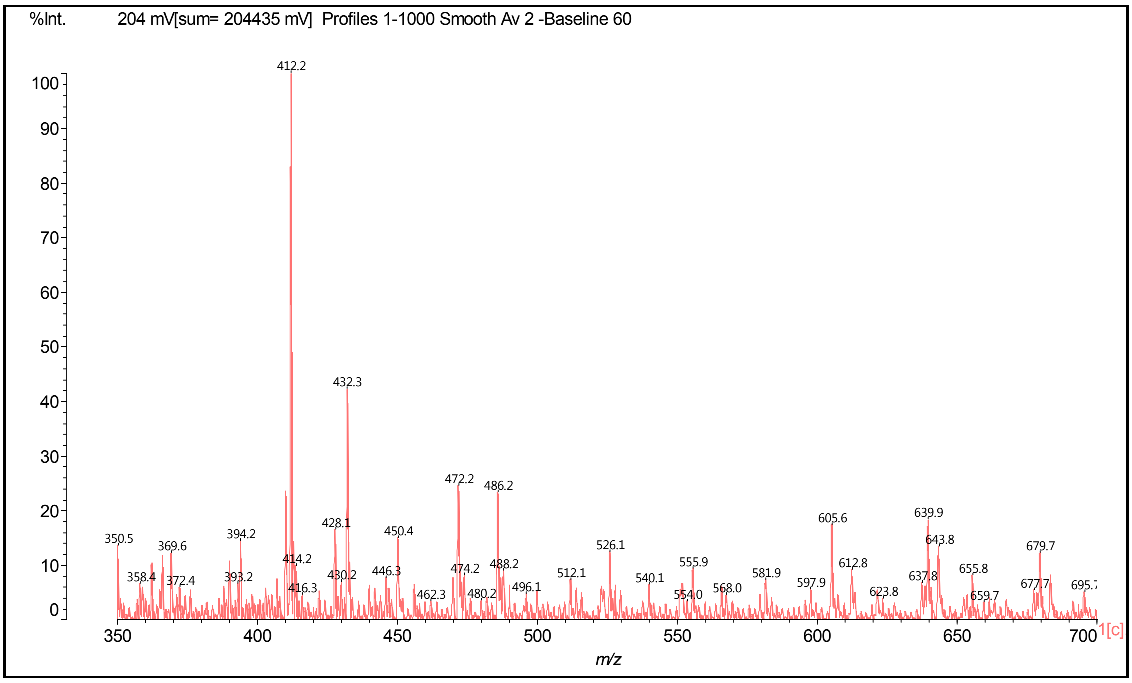

Figure S8. MALDI ToF spectrum of the reaction of mimosa + hexamethylenediamine + pTSA at 65 °C; 350–700 Da range.

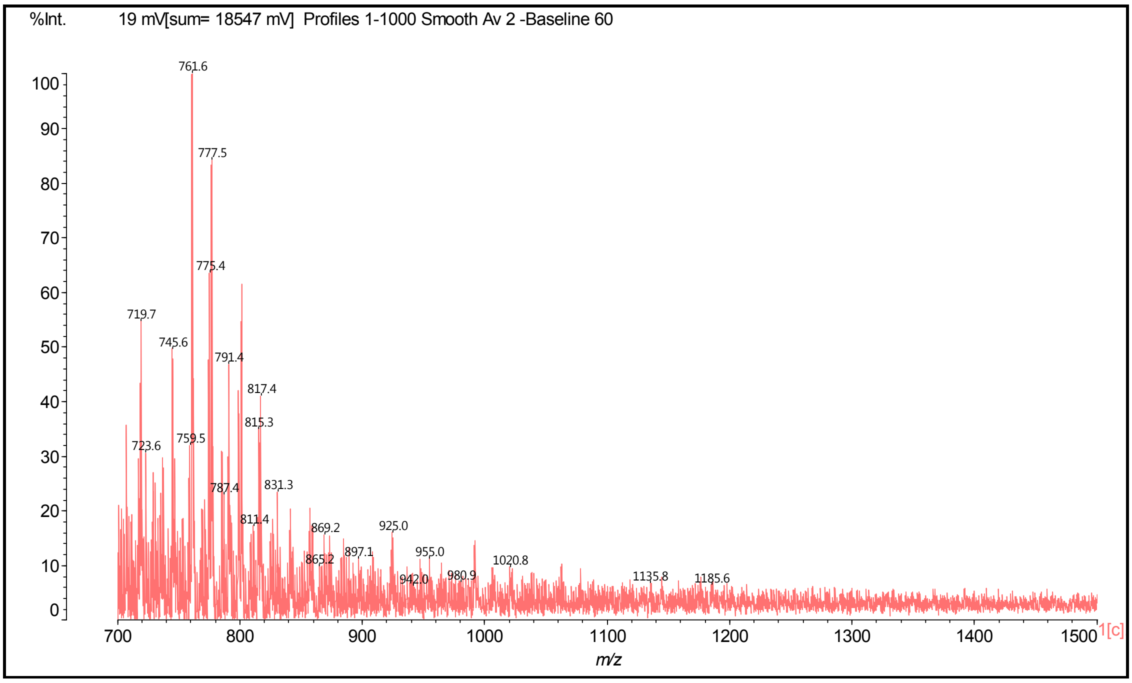

Figure S9. MALDI ToF spectrum of the reaction of mimosa + hexamethylenediamine + pTSA at 65 °C; 700–1500 Da range.

sAB65

Table S6. Structures determined by MALDI ToF of the uncatalysed reaction of mimosa + hexamethylenediamine at 65 °C.

Experimental	Calculated (Da)	Descrip. Cal (Da)	Description	Number Ionic Bonds	Number Covalent Bonds
390	390	274 + 116	F(−)(+)HMDA	1	-
410	410	290 − 17 + 114 + 23	F-HMDA(−)(+)Na	-	1
444	444	306 + 115 + 23	G(−)(+)HMDA(−)(+)Na	1	-
488	488	274 − 17 + 115 + 116	HMDA(+)(−)F-HMDA	1	1
468	468	274 + 114 × 2 − 17 × 2	(HMDA-F-HMDA) less 2H+	-	2
486	486	290 + 115 × 2 − 17 × 2	(HMDA-C-HMDA)	-	2
508	508	290 + 115 × 2 − 17 × 2 + 23 − 1	[C(HMDA)$_2$](−)(+)Na	-	2
524	524	306 + 114 + 115 − 17 − 17 + 23 − 1	(G[HMDA]$_2$)(−)(+)Na	-	2
526	525	290 + 114 + 115 − 17 + 23	HMDA-C(−)(+)HMDA(−)(+)Na	1	1
528	528	274 + 116 + 114 + 23	HMDA(+)(−)F(−)(+)HMDA(−)(+)Na	2	
560	560	306 + 116 × 2 + 23 − 1	[G((−)(+)HMDA)$_2$](−)(+)Na	2	-
566	568	274 + 115 × 3 − 17 − 17 − 17	F(HMDA)$_3$	-	3
580	578	288 + 288 + 2	Dimer	-	-
612	610	304 + 304 + 2	Dimer	-	-
619	618	306 + 115 × 2 − 17 × 2 + 116	HMDA(+)(−)G(HMDA)$_2$	1	2
621	622	306 + 115 × 2 + 114 − 17 × 3 + 23	[G(HMDA)$_3$](−)(+)Na	-	3
639	640	306 + 115 × 3 − 17 − 17 + 23	[HMDA(+)(−)G(HMDA)$_2$](−)(+)Na	1	2
643	644	274 + 116 × 3 + 23 − 1	[F((−)(+)HMDA)$_3$](−)(+)Na	3	-
643	644	272 + 272 + 2 + 114 − 17	F-F-HMDA	-	1
642	642	272 + 288 + 2 + 114 − 17 × 2	F-HMDA-C	-	2
653	654	306 + 116 × 3	G[(−)(+)HMDA]$_3$	3	-
661	660	272 + 288 + 2 + 115 − 17	F-C-HMDA	-	1
661	662	272 + 272 + 2 + 116	F-F(−)(+)HMDA	1	-
677	676	306 + 116 × 2 + 115 + 23	[HMDA(+)(−)]$_2$ G (−)(+)HMDA(−)(+)Na	3	-
679	678	272 + 288 + 2 + 116	F-C(−)(+)HMDA	1	-
683	682	272 + 288 + 2 + 114 − 17 + 23	[F-C-HMDA]	-	1
695	694	288 + 288 + 2 + 116	C-C(−)(+)HMDA	1	-
719	717	288 + 288 + 2 + 116 + 23	[C-C(−)(+)HMDA](−)(+)Na	1	-
723	723	272 + 272 + 2 + 114 + 115 − 17 × 3	F-HMDA-F-HMDA	-	3
723	724	274 + 116 × 4 − 18 × 2 + 23 − 1	[(HMDA)$_2$F((−)(+)HMDA)$_2$](−)(+)Na	2	2
759	758	272 + 288 + 2 + 115 × 2 − 17 × 2	HMDA-F-C-HMDA	-	2
761	760	274 + 290 + 114 − 17 × 2 + 116	F-HMDA-C(−)(+)HMDA	1	2
761	760	272 + 272 + 2 + 115 − 17 + 116	HMDA-F-F(−)(+)HMDA	1	1
777	776	272 + 288 + 2 + 115 − 17 + 116	HMDA-F-C(−)(+)HMDA	1	1
857	857	272 + 272 + 288 + 2 + 23	Trimer	-	-
1366	1368	288 × 2 + 2 + 116 × 2 − 18 × 3 + 304 × 2 + 2 (Or 288 × 4 + 2 + 116 × 2 − 18)	C-G-HMDA-C-G-HMDA or HMDA-C-C-C-C(−)(+)HMDA	-	3
1480	1482	288 × 2 + 304 × 2 + 2 + 116 × 3 − 18 × 3	(G-C-C-G)(HMDA)$_3$	-	3

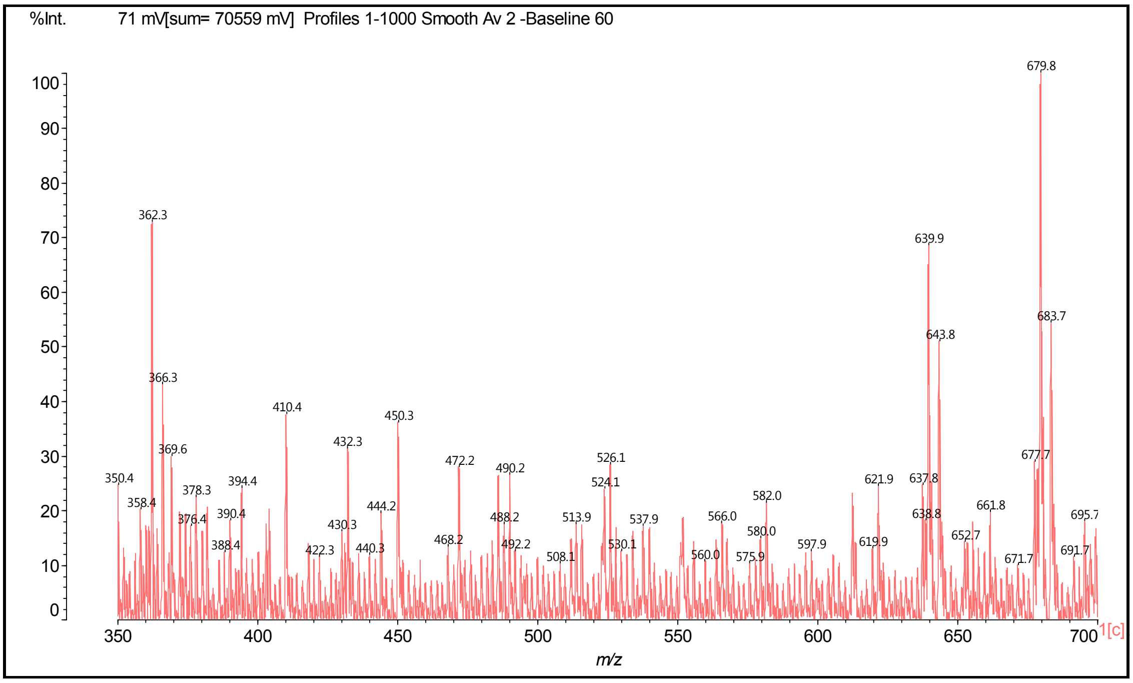

Figure S10. MALDI ToF spectrum of the uncatalysed reaction of mimosa + hexamethylenediamine at 65 °C; 350–700 Da range.

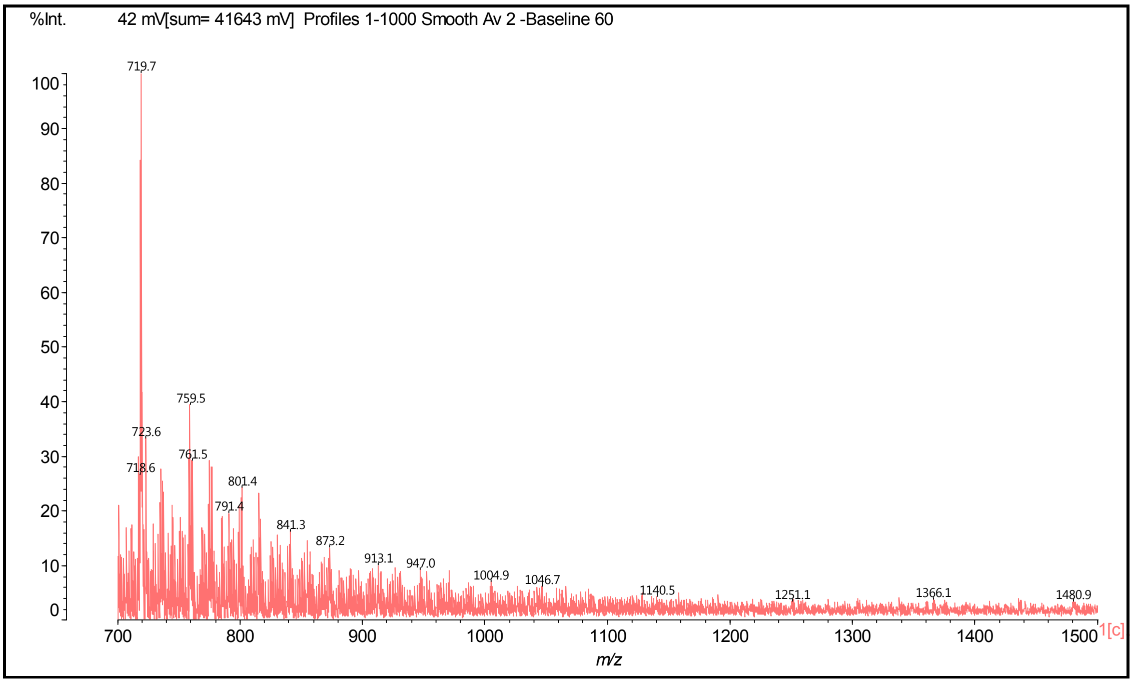

Figure S11. MALDI ToF spectrum of the uncatalysed reaction of mimosa + hexamethylenediamine at 65 °C; 700–1500 Da range.

2. Catechin Model Compound Reactions

[M+Na+] = Cat (288 Da) + HMDA (114 Da) + 2H (end group) + 23 Na+
Cat = catechin
HMDA = hexamethylenediamine

sAB185

Table S7. Structures determined by MALDI ToF of the uncatalysed reaction of catechin monomer + hexamethylenediamine at 185 °C.

Experimental [Mn + Na+] (on the Graph) (Da)	Calculated [Mn + Na+] (Da)	Description
23.1	23	Na+
39.1	35.5	Cl−
117	116	HMDA
137	139	HMDA + Na+
289.9	290	Cat
315.8	313	Cat + Na+
370.7	367	3× HMDA + Na+
401.8	404	Cat + HMDA
428.8	427	Cat + HMDA + Na+
522.5	518	HMDA + Cat + HMDA
544.5	541	HMDA + Cat + HMDA + Na+
698.6	692	Cat + HMDA + Cat
711.4	709	6× HMDA + Na+
888.8	889	Cat + Cat + Cat + Na+
1103.2	1096	Cat + HMDA + Cat + HMDA + Cat
1220.5	1208	Cat + HMDA + Cat + HMDA + Cat + HMDA
1279.4	1268	Cat + Cat + HMDA + Cat + Cat
1397.6	1405	Cat + HMDA + Cat + Cat + HMDA + Cat + Na+
1454.7	1459	HMDA +Cat + HMDA + Cat + HMDA + Cat + HMDA + HMDA + Na+
1513.9	1519	Cat + HMDA + Cat + HMDA + Cat +HMDA + Cat + Na+

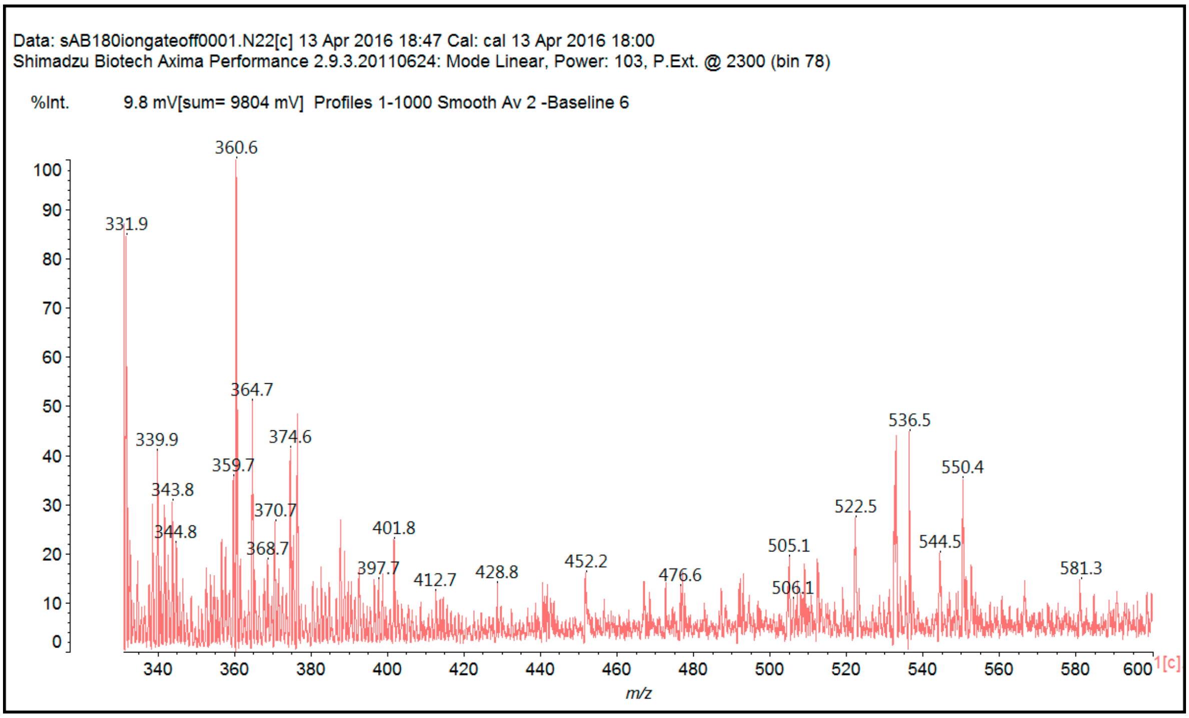

Figure S12. MALDI ToF of the uncatalysed reaction of catechin monomer + hexamethylenediamine at 185 °C; 330–600 Da range.

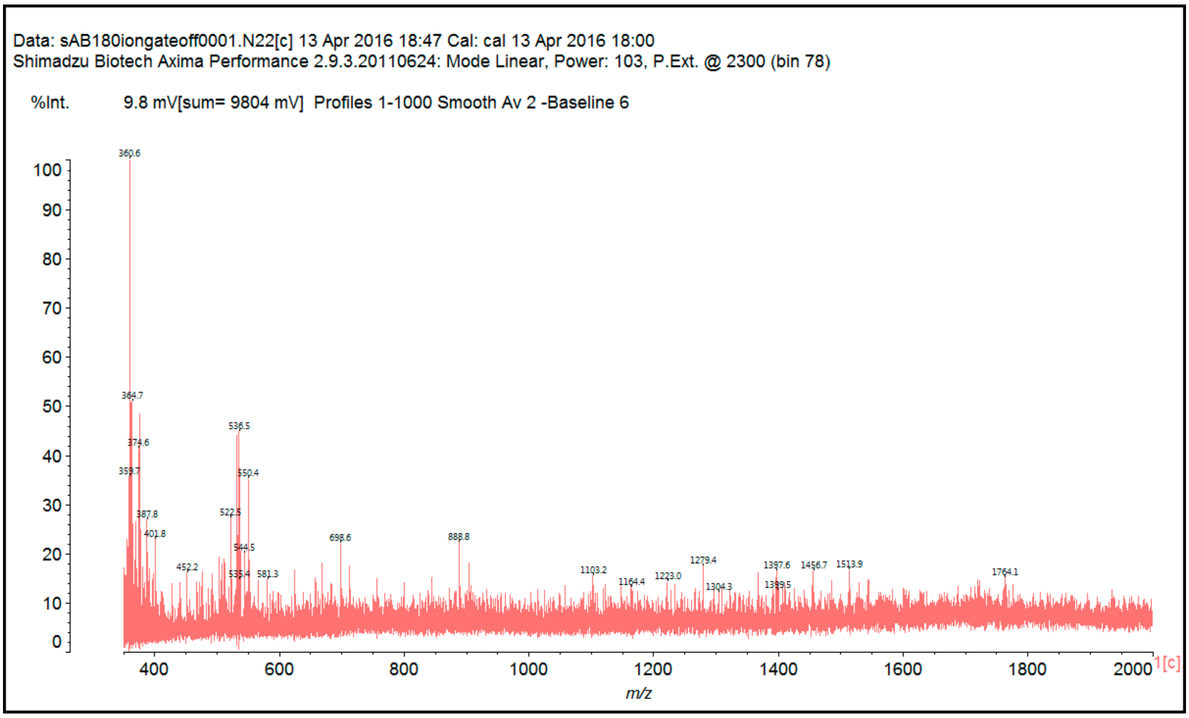

Figure S13. MALDI ToF of the uncatalysed reaction of catechin monomer + hexamethylenediamine at 185 °C; 350–2000 Da range.

sAB100

Table S8. Structures determined by MALDI ToF of the uncatalysed reaction of catechin monomer + hexamethylenediamine at 100 °C.

Experimental [Mn + Na+] (on the Graph) (Da)	Calculated [Mn + Na+] (Da)	Description
23.0	23	Na^+
38.9	35.5	Cl^-
117.2	116	HMDA
137.1	139	HMDA + Na^+
289.9	290	Cat
314.7	313	Cat + Na^+
368.6	367	3 × HMDA + Na^+
424.7	427	Cat + HMDA + Na^+
520.8	518	HMDA + Cat + HMDA
540.8–546.6	541	HMDA + Cat + HMDA + Na^+
719.6	715	Cat + HMDA + Cat + Na^+
893	889	Cat + Cat + Cat + Na^+
1087	1094	Cat + HMDA + Cat + HMDA + Cat
1221.7	1208	Cat + HMDA + Cat + HMDA + Cat + HMDA
1338.8	1345	HMDA + Cat + HMDA + Cat + HMDA + Cat + HMDA + Na^+
1397.3	1382	Cat + HMDA + Cat + Cat + HMDA + Cat
1455.2	1459	HMDA + Cat + HMDA + Cat + HMDA + Cat + HMDA + HMDA + Na^+
1746.7	1745	HMDA + Cat + HMDA + Cat + HMDA + Cat + HMDA + Cat + HMDA + Na^+

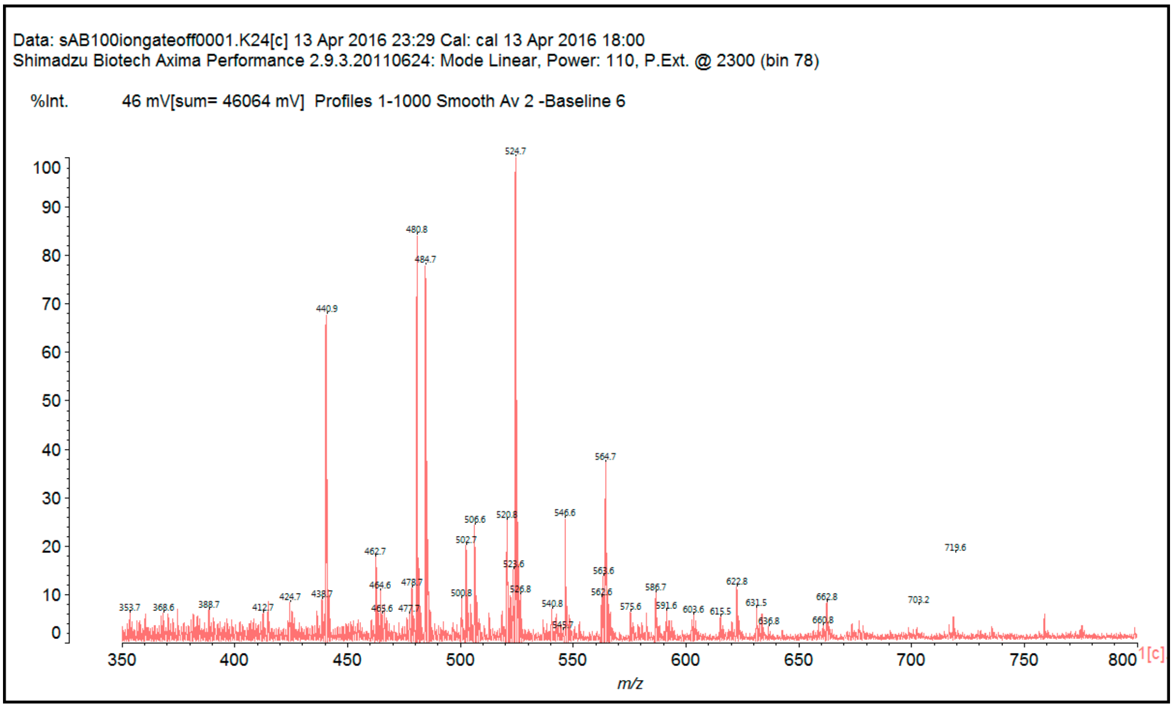

Figure S14. MALDI ToF spectrum of the uncatalysed reaction of catechin monomer + hexamethylenediamine at 100 °C; 350–800 Da range.

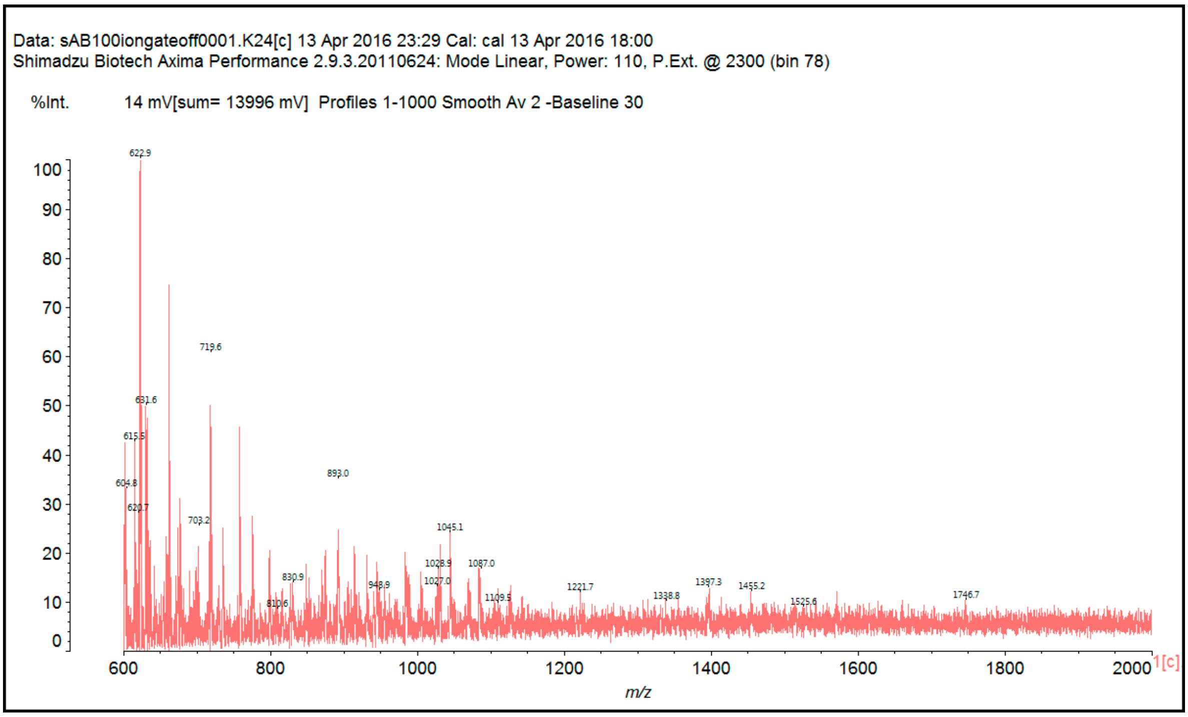

Figure S15. MALDI ToF spectrum of the uncatalysed reaction of catechin monomer + hexamethylenediamine at 100 °C; 600–2000 Da range.

sAB65

Table S9. Structures determined by MALDI ToF of the uncatalysed reaction of catechin monomer + hexamethylenediamine at 65 °C.

Experimental [Mn + Na+] (on the Graph) (Da)	Calculated [Mn + Na+] (Da)	Description
23.1	23	Na^+
39.1	35.5	Cl^-
117.2	116	HMDA
137	139	HMDA + Na^+
280	290	Cat
317	313	Cat + Na^+
344.9	344	3× HMDA
368.1	367	3 × HMDA + Na^+
399.1	404	Cat + HMDA
428.8	427	Cat + HMDA + Na^+
522.9	518	HMDA + Cat + HMDA
542.9	541	HMDA + Cat + HMDA + Na^+
576.9	572	5 × HMDA
702	692	Cat + HMDA + Cat
811.9	806	Cat + HMDA + Cat + HMDA
895	889	Cat + Cat + Cat + Na^+
919	922	HMDA + Cat + HMDA + Cat + HMDA
1033.2	1028	9 × HMDA

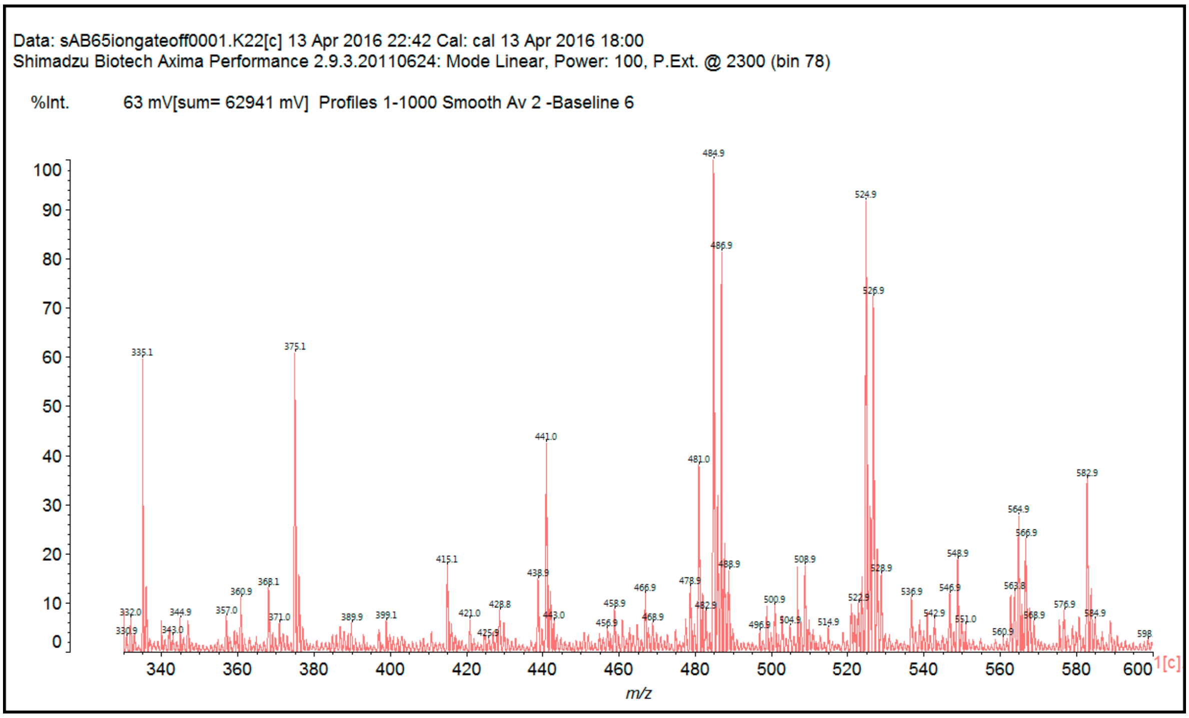

Figure S16. MALDI ToF spectrum of the uncatalysed reaction of catechin monomer + hexamethylenediamine at 65 °C; 330–600 Da range.

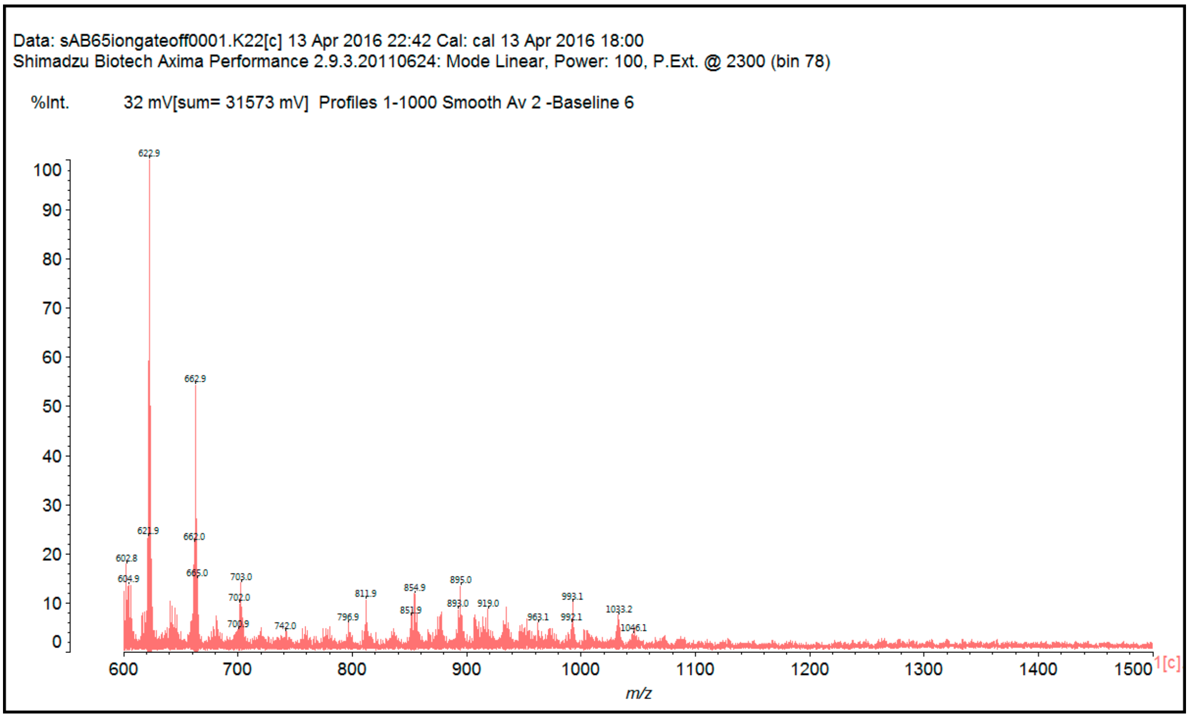

Figure S17. MALDI ToF spectrum of the uncatalysed reaction of catechin monomer + hexamethylenediamine at 65 °C; 600–1500 Da range.

B180

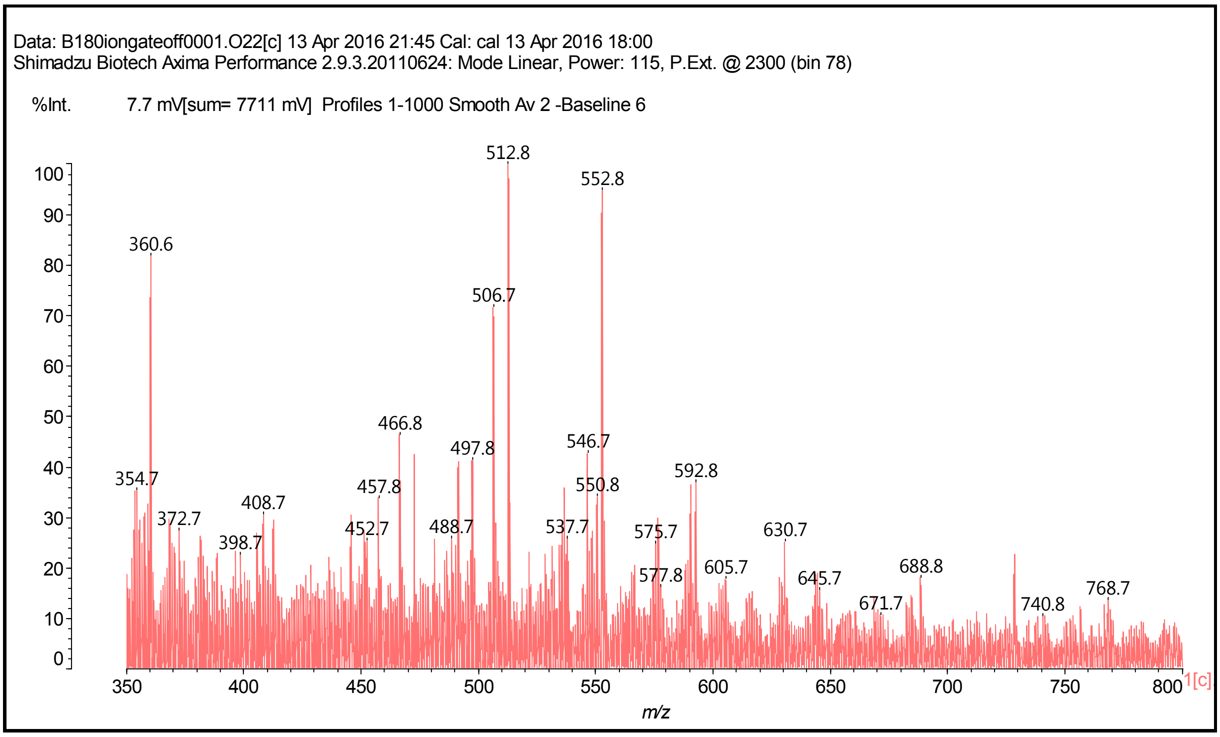

Figure S18. MALDI ToF spectrum of the reaction of catechin monomer + hexamethylenediamine + NaOH at 185 °C; 350–800 Da range.

B180

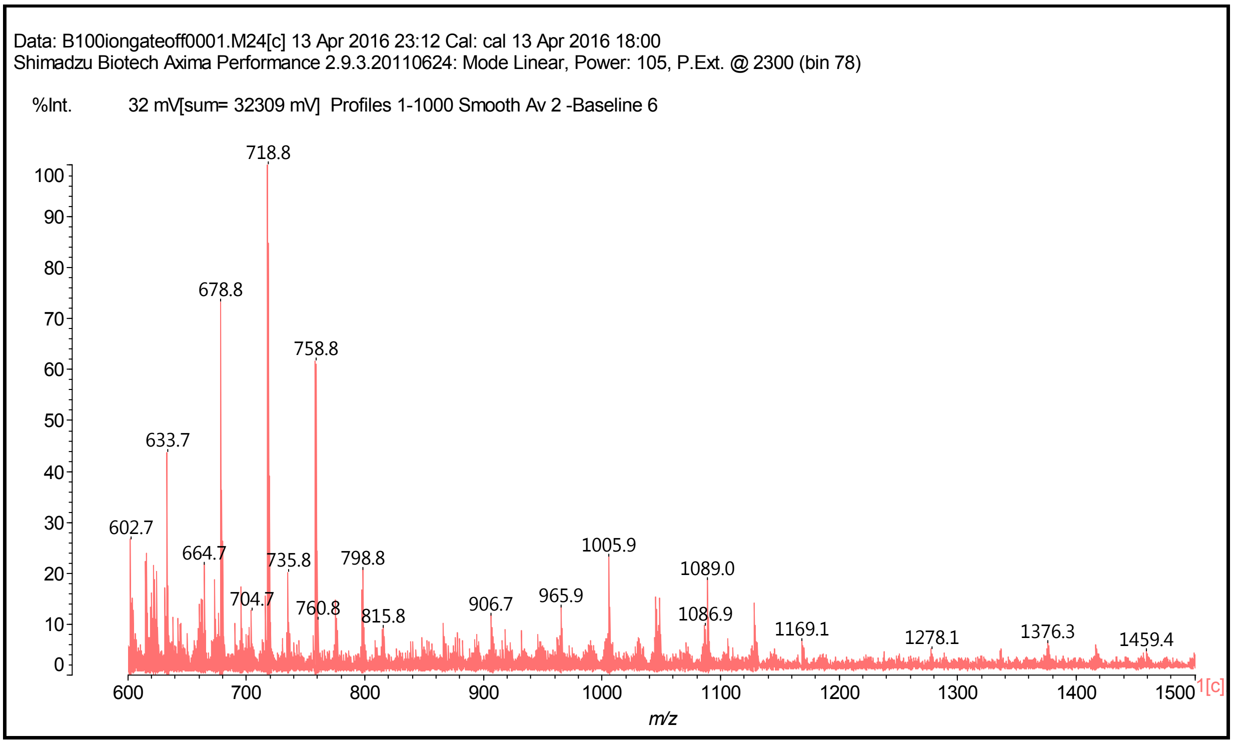

Figure S19. MALDI ToF spectrum of the reaction of catechin monomer + hexamethylenediamine + NaOH at 185 °C; 600-1500 Da range.

B100

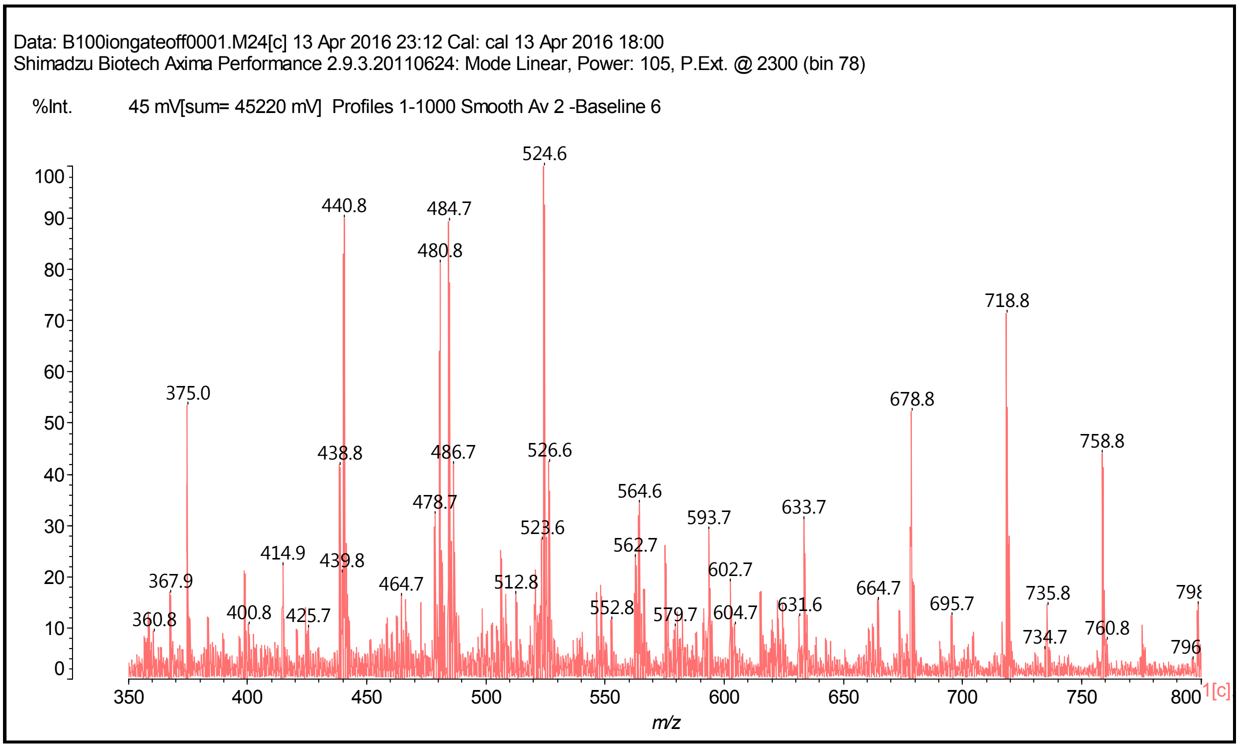

Figure S20. MALDI ToF spectrum of the reaction of catechin monomer + hexamethylenediamine + NaOH at 100 °C; 350–800 Da range.

B100

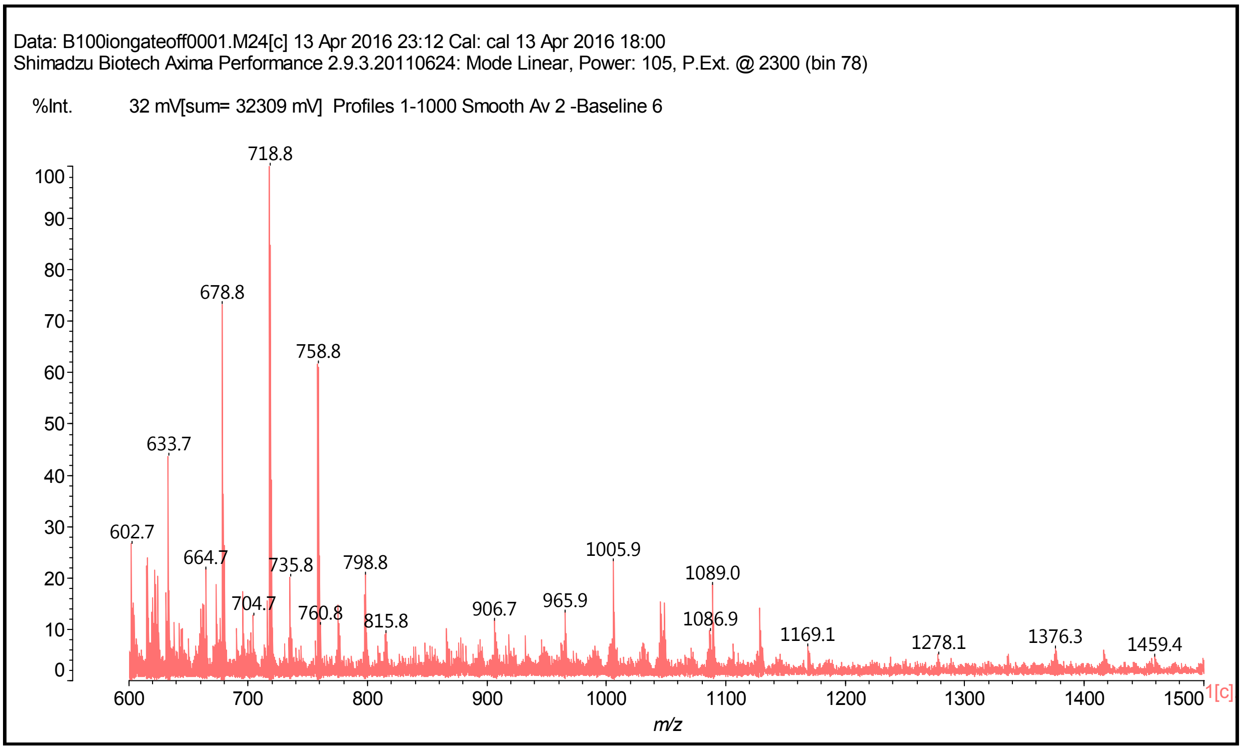

Figure S21. MALDI ToF spectrum of the reaction of catechin monomer + hexamethylenediamine + NaOH at 100 °C; 600 – 1500 Da range.

B65

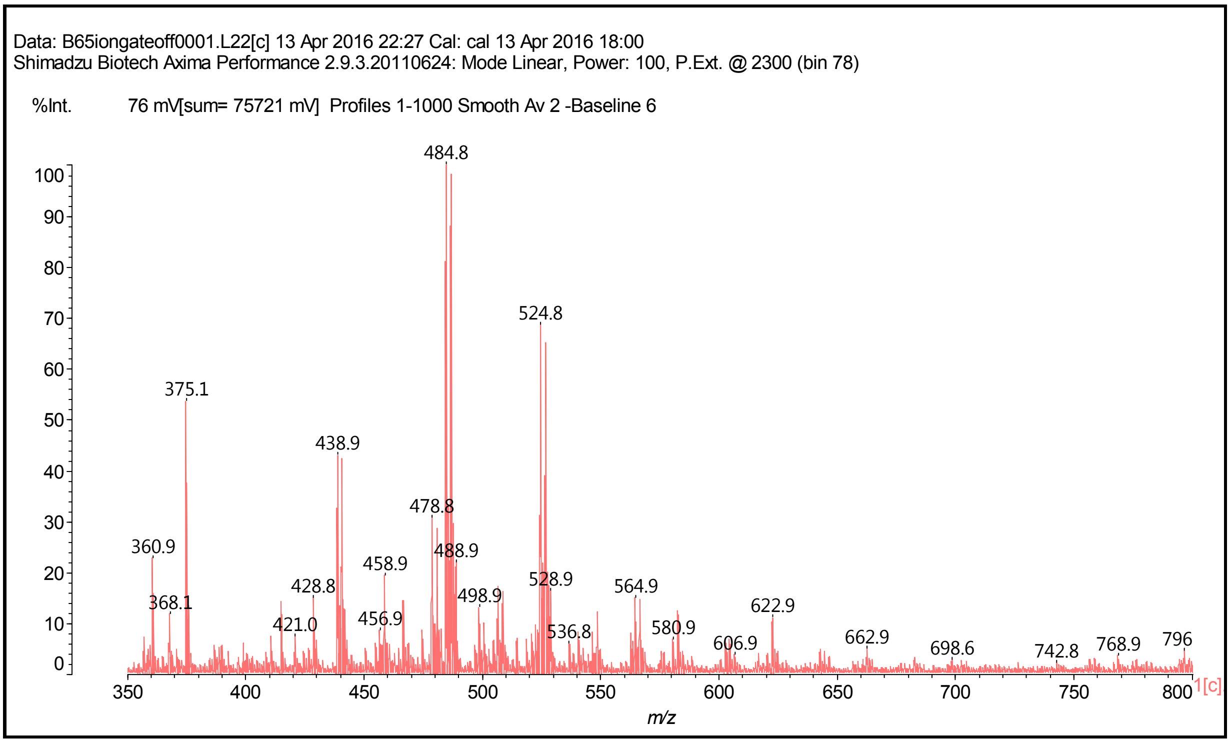

Figure S22. MALDI ToF spectrum of the reaction of catechin monomer + hexamethylenediamine + NaOH at 65 °C; 350–800Da range.

B65

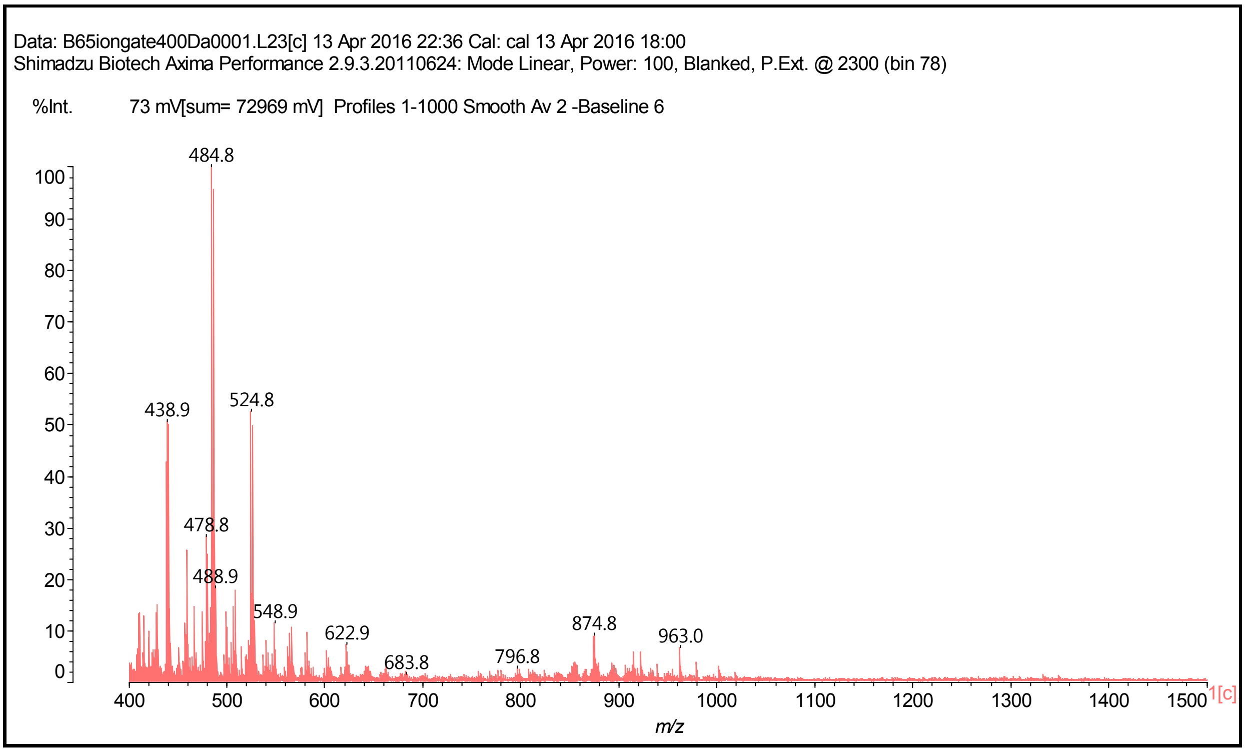

Figure S23. MALDI ToF spectrum of the reaction of catechin monomer + hexamethylenediamine + NaOH at 65 °C; 600–1500 Da range.

A180

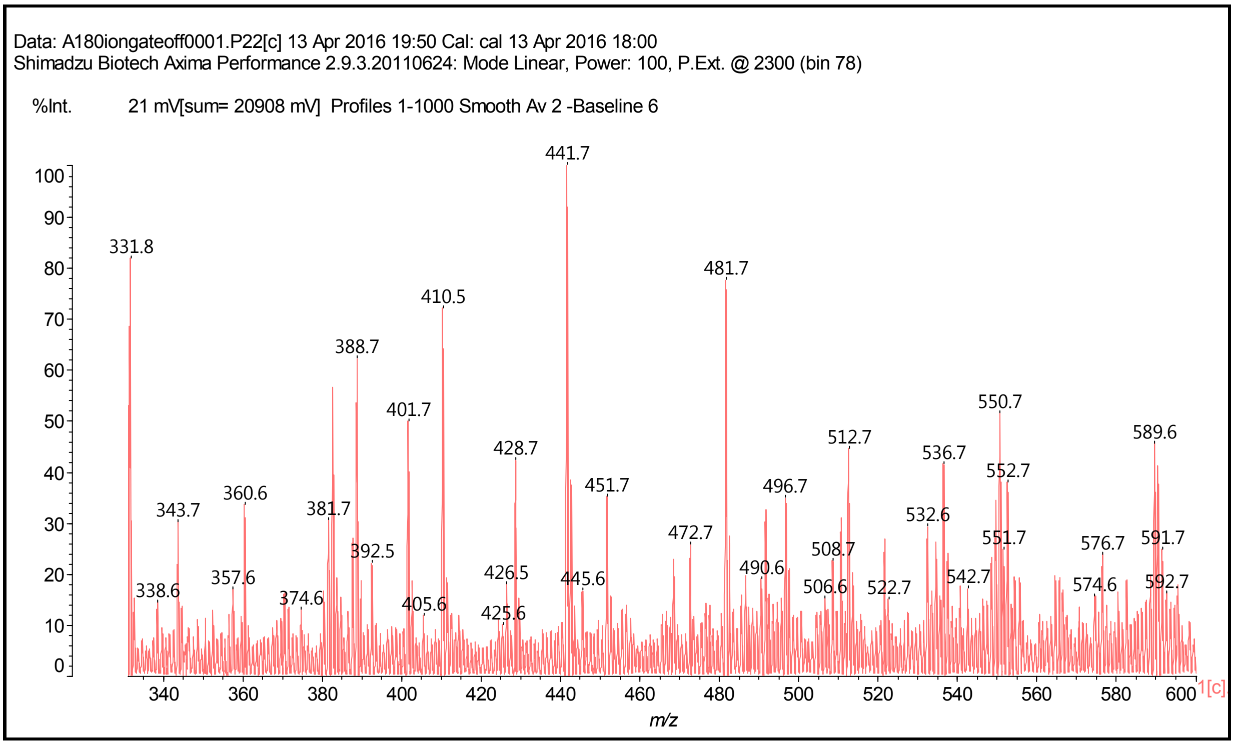

Figure S24. MALDI ToF spectrum of the reaction of catechin monomer + hexamethylenediamine + pTSA at 185 °C; 350-800 Da range.

A100

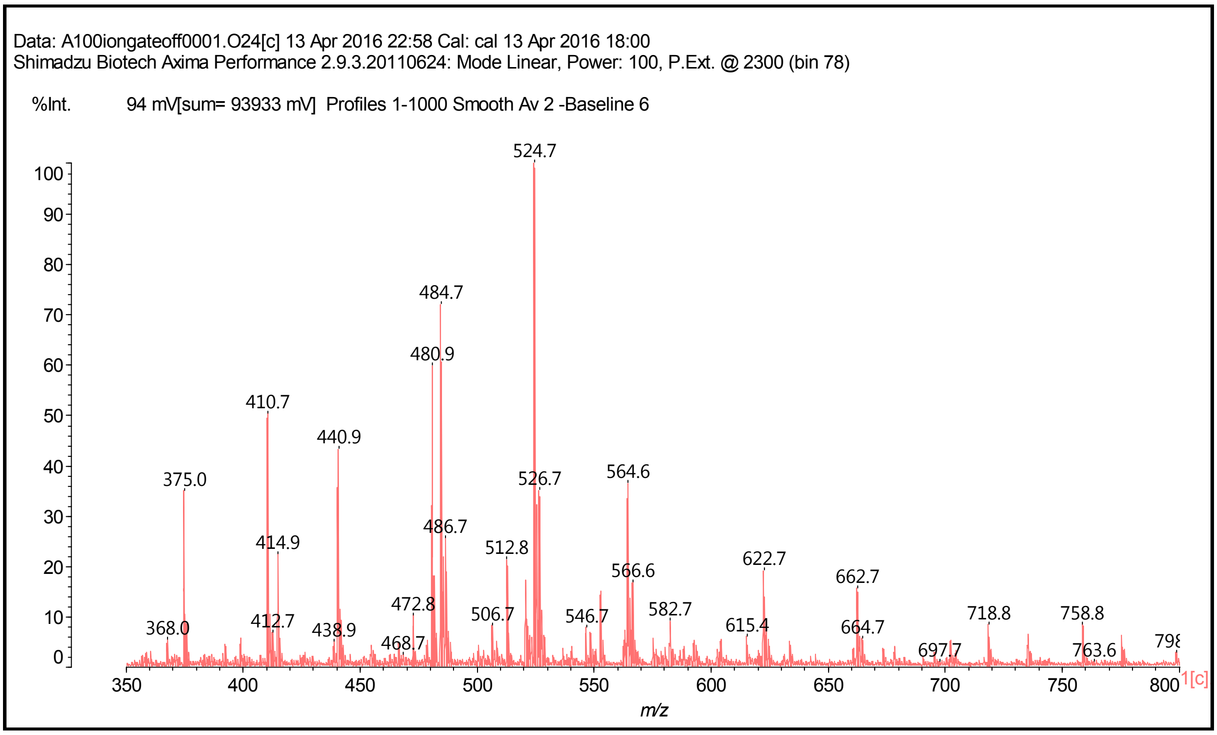

Figure S25. MALDI ToF spectrum of the reaction of catechin monomer + hexamethylenediamine + pTSA at 100 °C. 350–800 Da range.

A100

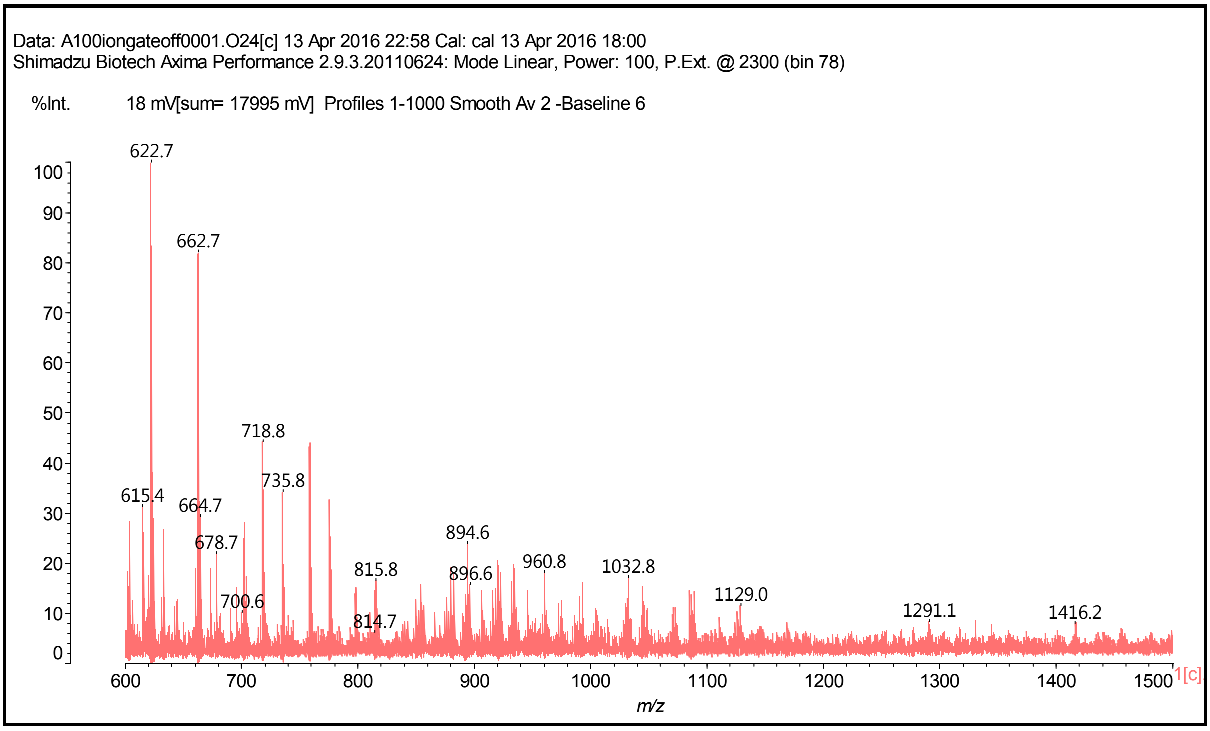

Figure S26. MALDI ToF spectrum of the reaction of catechin monomer + hexamethylenediamine + pTSA at 100 °C; 600–1500 Da range.

A65

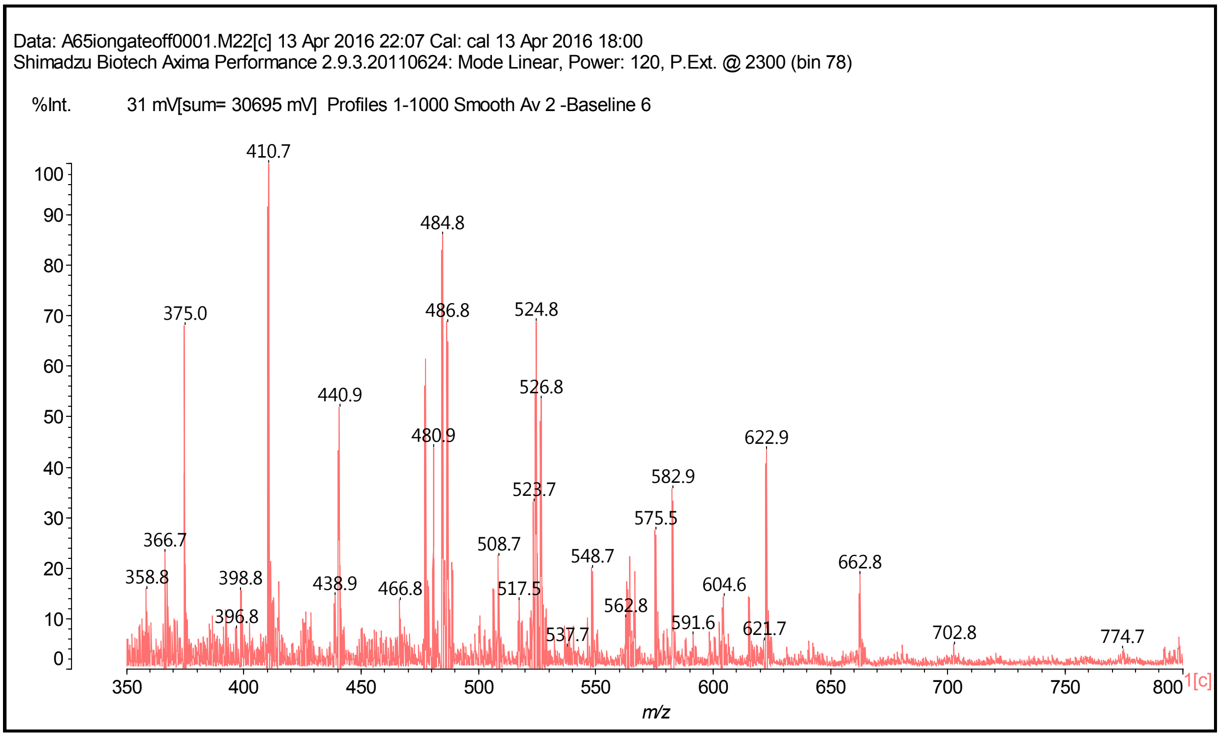

Figure S27. MALDI ToF spectrum of the reaction of catechin monomer + hexamethylenediamine + pTSA at 65 °C; 350–800 Da range.

A65

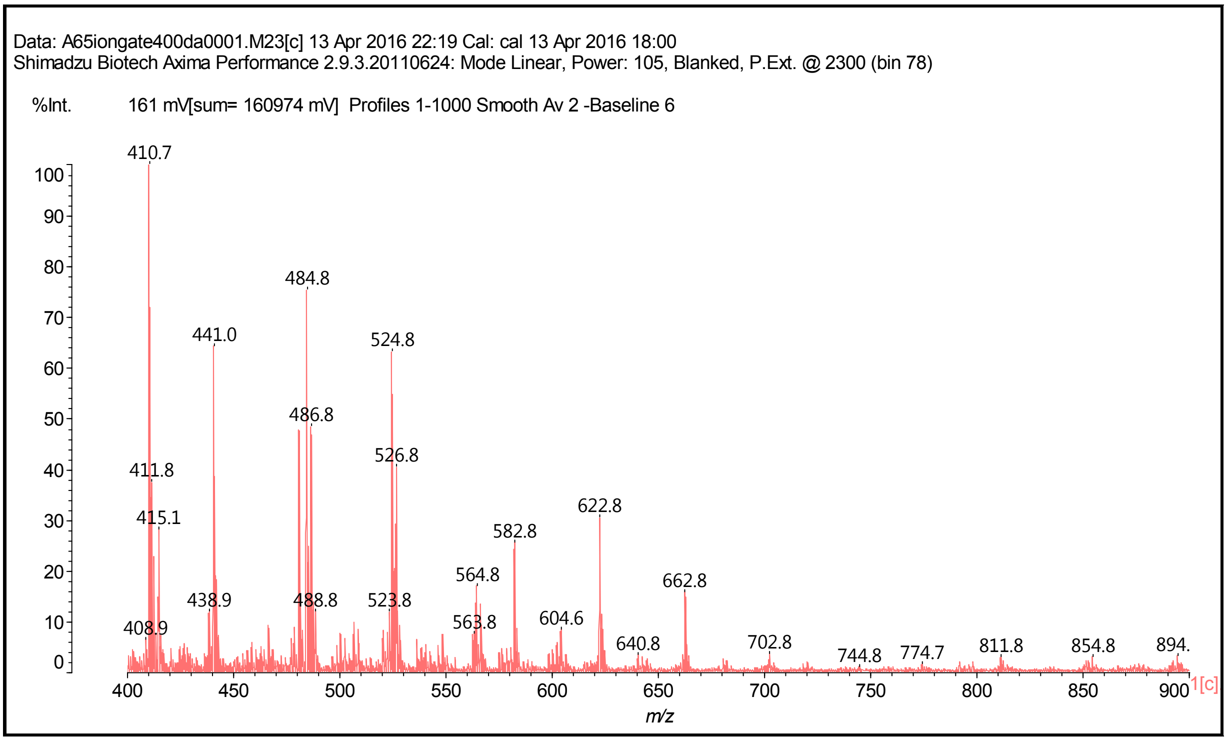

Figure S28. MALDI ToF spectrum of the reaction of catechin monomer + hexamethylenediamine + pTSA at 65 °C; 400–900 Da range.

CP MAS ^{13}C NMR

Catechin A180

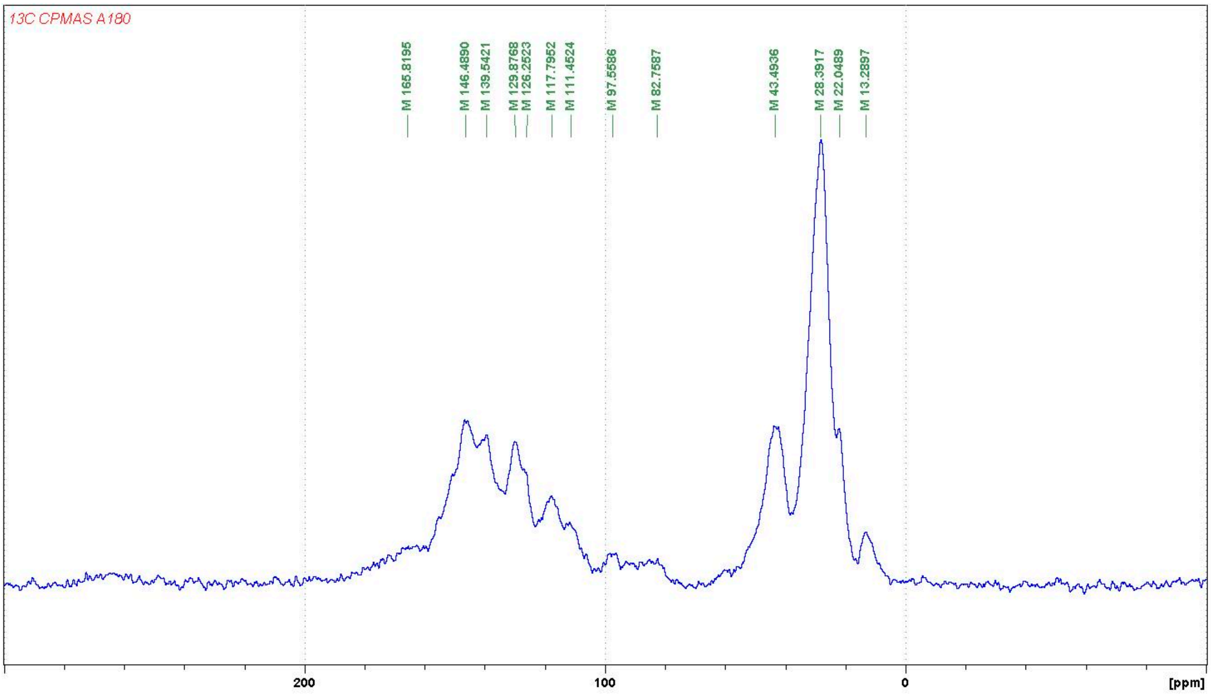

Figure S29. CP MAS ^{13}C NMR spectrum of the reaction of catechin monomer + hexamethylenediamine + pTSA at 185 °C.

Catechin A 100

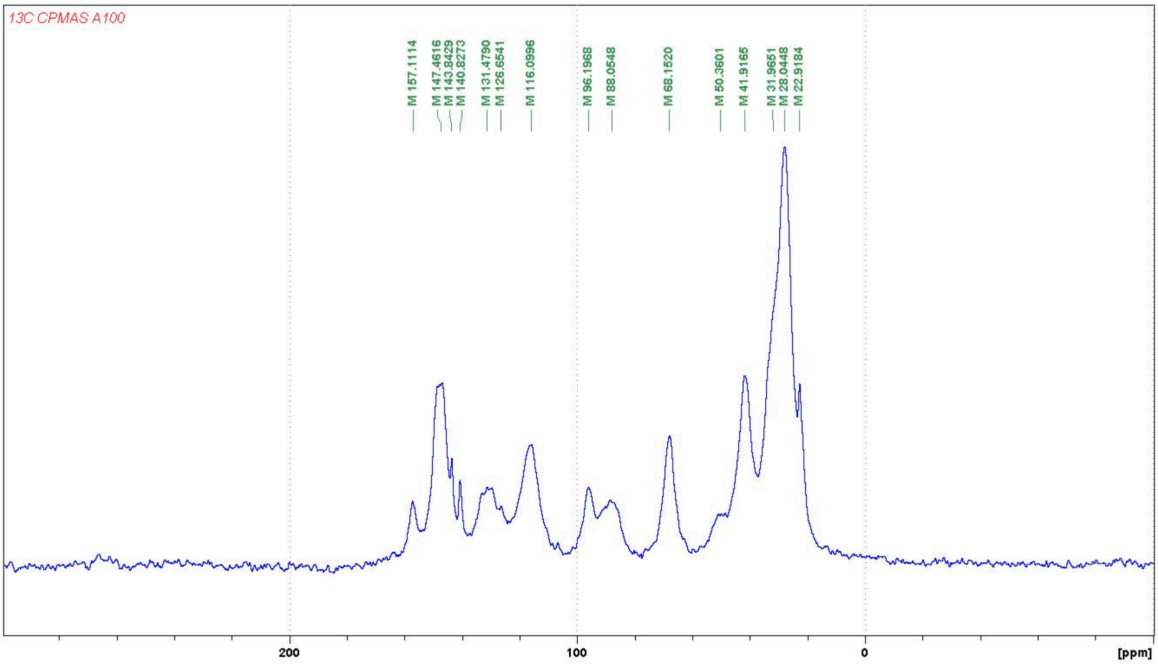

Figure S30. CP MAS ^{13}C NMR spectrum of the reaction of catechin monomer + hexamethylenediamine + pTSA at 100 °C.

Catechin B180

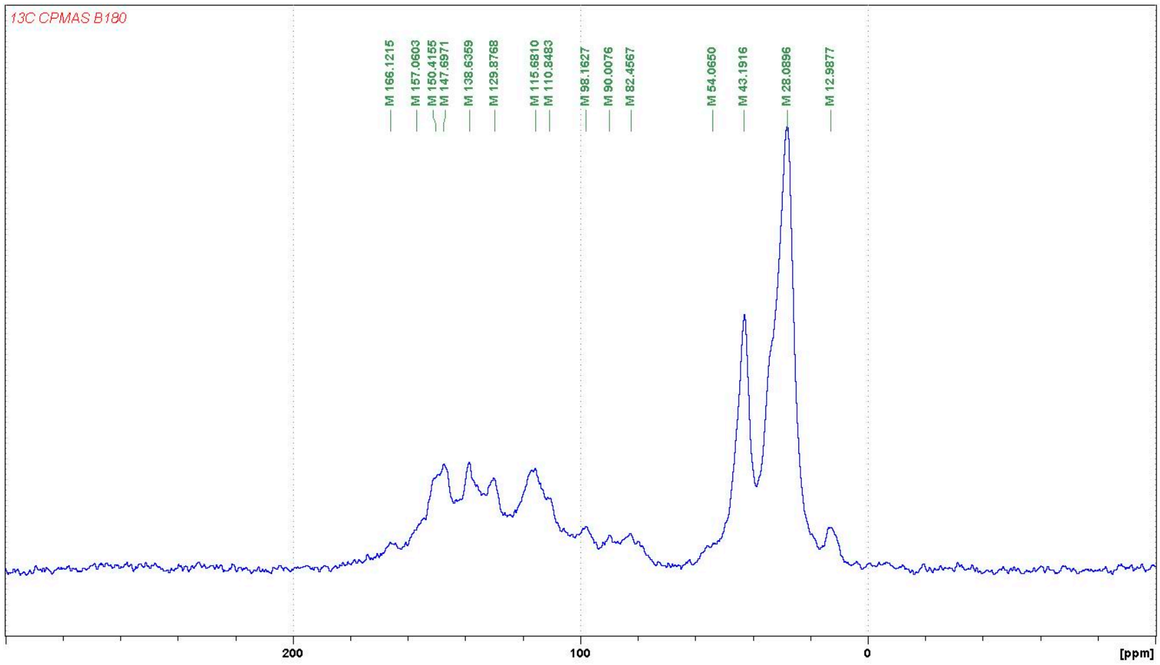

Figure S31. CP MAS ^{13}C NMR spectrum of the reaction of catechin monomer + hexamethylenediamine + NaOH at 185 °C.

Catechin B100

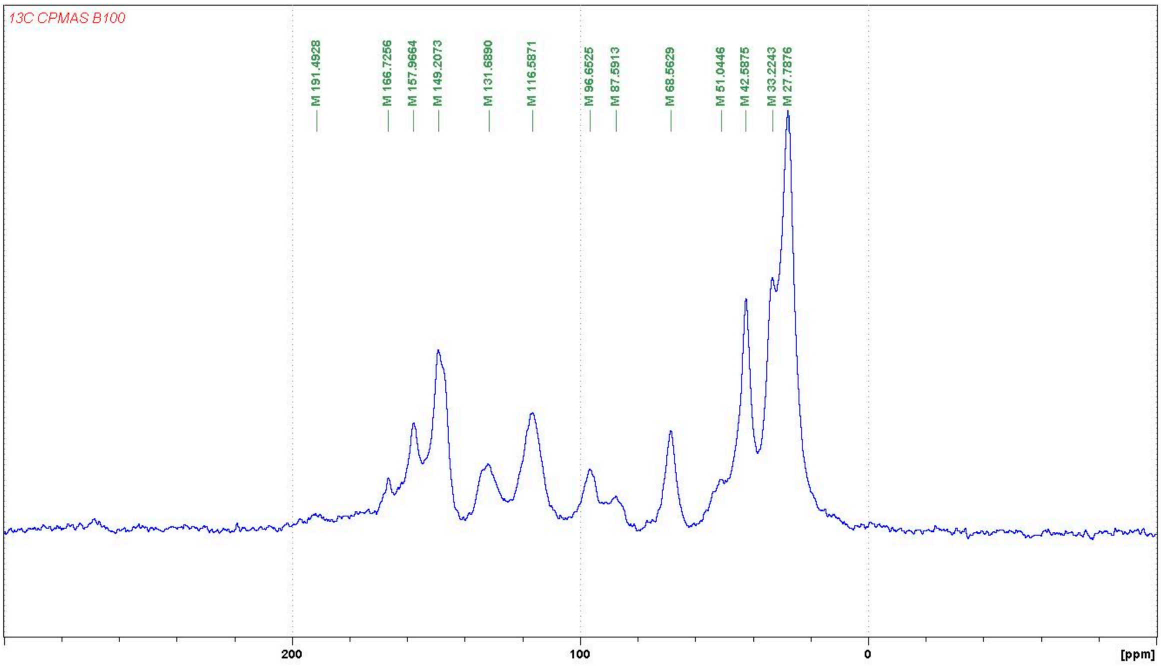

Figure S32. CP MAS ^{13}C NMR spectrum of the reaction of catechin monomer + hexamethylenediamine + NaOH at 100 °C.

Catechin SAB 180

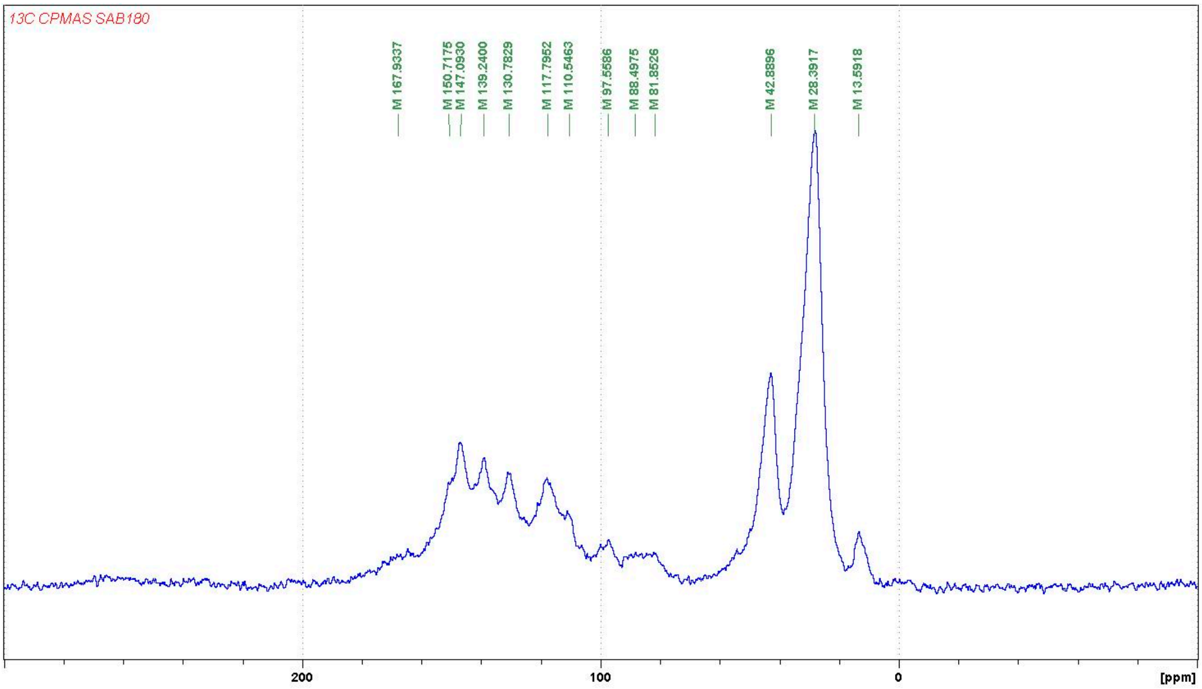

Figure S33. CP MAS ^{13}C NMR spectrum of the uncatalysed reaction of catechin monomer + hexamethylenediamin at 185 °C.

Catechin SAB100

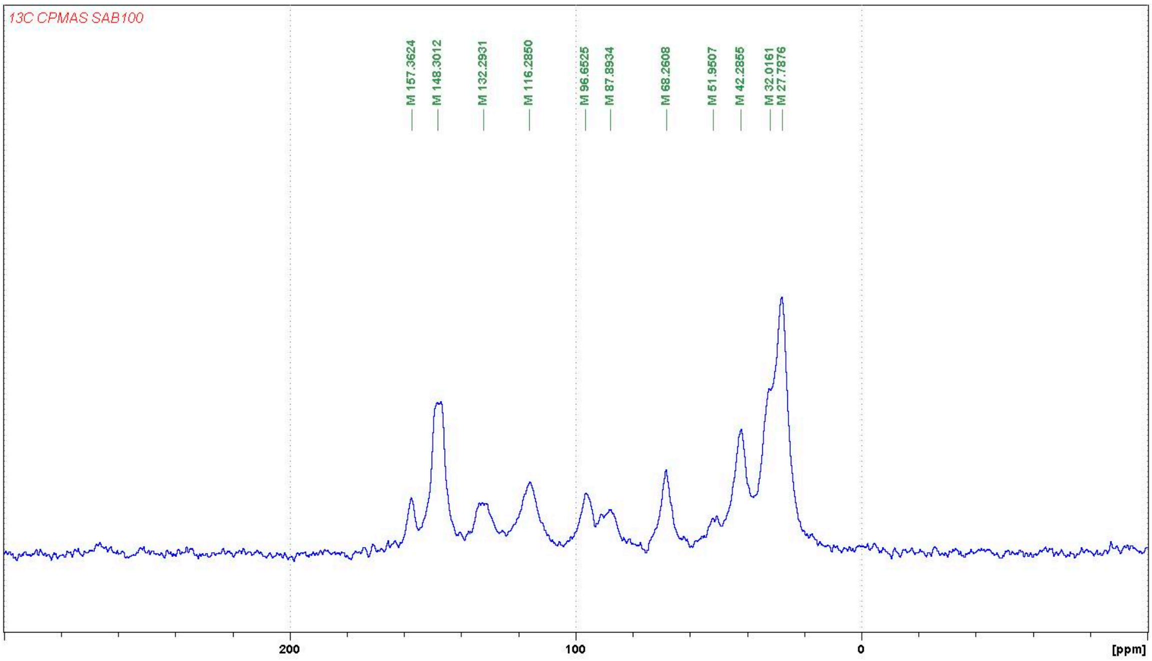

Figure S34. CP MAS ^{13}C NMR spectrum of the uncatalysed reaction of catechin monomer + hexamethylenediamin at 100 °C.
